# Activity-based probes and chemical proteomics uncover the biological impact of targeting HMG-CoA Synthase 1 in the mevalonate pathway

**DOI:** 10.1016/j.jbc.2025.110660

**Published:** 2025-09-03

**Authors:** Sang Ah Yi, Liang Sun, Yi Rao, Alban Ordureau, Jason S. Lewis, Heeseon An

**Affiliations:** 1 Chemical Biology Program, Sloan Kettering Institute, Memorial Sloan Kettering Cancer Center, New York, New York, USA; 2 School of Pharmacy, Sungkyunkwan University, Suwon, Republic of Korea; 3 Department of Biopharmaceutical Convergence, Sungkyunkwan University, Suwon-si, Republic of Korea; 4 Department of Radiology, Memorial Sloan Kettering Cancer Center, New York, New York, USA; 5 Molecular Pharmacology Program, Sloan Kettering Institute, Memorial Sloan Kettering Cancer Center, New York, New York, USA; 6 Department of Pharmacology, Weill Cornell Graduate School of Medical Sciences, New York, New York, USA; 7 Cell Biology Program, Sloan Kettering Institute, Memorial Sloan Kettering Cancer Center, New York, New York, USA

**Keywords:** HMGCS1, mevalonate, hymeglusin, HMGCR, statin, chemical proteomics, activity-based probe

## Abstract

Mevalonate is a precursor for essential metabolites, such as isoprenoids and sterols. Its synthesis starts with 3-hydroxy-3-methylglutaryl-coenzyme A synthase 1 (HMGCS1) producing HMG-CoA, which is then converted to mevalonate by HMG-CoA reductase (HMGCR), a target of statins. Cancer cells often upregulate enzymes in the mevalonate pathway to meet their metabolic demands, leading to the development of inhibitors targeting several enzymes in this pathway. However, current inhibitors have not yet shown significant anticancer activity. While HMGCS1 has unique biochemical properties that distinguish it from other mevalonate pathway enzymes, the effects of inhibiting HMGCS1 have not been thoroughly investigated. Here, we present a set of chemical probes that enable us to systematically assess the proteome-wide selectivity and potency of Hymeglusin, the primary inhibitor of HMGCS1 used in the field, confirming it as a useful tool for short-term HMGCS1 inhibition. Inhibiting HMGCS1 with Hymeglusin causes proteome changes that are nearly identical to those caused by inhibiting HMGCR or degrading HMGCS1. Accordingly, simultaneously targeting HMGCS1 and HMGCR effectively suppresses the growth of statin-resistant cells and xenograft models, without increasing the risk of side effects. Finally, we find that while Hymeglusin is a valuable tool for short-term mechanistic studies, its usefulness is limited for long-term efficacy studies due to its poor stability in serum. Together, this study highlights the biological implications of targeting HMGCS1 as monotherapy or in combination with statins, and caution is required when using Hymeglusin as a tool.

Mevalonate is an essential precursor for the biosynthesis of sterols, isoprenoids, vitamin D, and ubiquinone (1, 2). Consequently, the enzymes involved in the mevalonate pathway (MVP) play crucial roles in multiple aspects of cellular physiology, from membrane biology to cell signaling and mitochondrial metabolism (3). The synthesis of mevalonate begins with the first step, catalyzed by 3-hydroxy-3-methylglutaryl-coenzyme A synthase 1 (HMGCS1), which condenses acetyl-CoA with acetoacetyl-CoA to produce HMG-CoA. The HMG-CoA then undergoes reduction to mevalonate *via* HMG-CoA reductase (HMGCR). Following this, approximately 20 downstream enzymes convert mevalonate into sterols and other nonsterol end products. Growing evidence indicates that cancer cells exhibit upregulation of MVP enzymes, which may stimulate glucose uptake and various signaling pathways, including Ras, Rab, and Rho pathways (4, 5, 6, 7, 8). This upregulation has also been linked to resistance to various chemotherapies, emphasizing the significance of these enzymes in cancer progression and their ability to manage stress (9, 10, 11). Prior research suggests that inhibiting the MVP induces apoptotic death of cancer cells by blocking the geranylgeranylation of proteins (12, 13). Consequently, several enzymes in this pathway—such as HMGCR, farnesyl pyrophosphate synthase, geranylgeranyl pyrophosphate synthase, farnesyltransferase, and geranylgeranyltransferase—have been exploited as potential pharmacological targets (5, 7). However, the existing approaches or inhibitors have yet to produce significant clinical anticancer activity (7, 14, 15, 16, 17, 18, 19, 20, 21).

HMGCS1 represents an underexplored pharmacological target within the MVP, despite the fact that it possesses several attractive features that distinguish it from HMGCR and other downstream MVP enzymes. Notably, HMGCS1 contains a catalytic cysteine in its active site that can be readily leveraged for developing covalent inhibitors and chemical probes to enhance potency and sustained efficacy compared to noncovalent inhibitors. Moreover, we previously demonstrated that HMGCS1 levels are directly regulated by the master regulator of cell growth, mechanistic target of rapamycin (mTORC1) (22). Studies have reported increased demand for HMGCS1 during active cell proliferation in cancer cells, with 60% of them showing hyperactive mTORC1 signaling, potentially making it a limiting enzyme for the progression of many cancer cells (22, 23, 24). Thus, perturbation of HMGCS1 can be an alternative strategy for treating certain type of cancers (22).

However, the chemical tools available to perturb the HMGCS1 activity are currently limited, with Hymeglusin (HG) serving as the primary small molecule probe (10, 25, 26). Identified as an HMGCS1 inhibitor in 1987, HG was isolated from Fusarium and Scopulariopsis fungi (27, 28, 29, 30, 31, 32). The cocrystal structure of HMGCS1 and HG revealed that HG forms a thioester bond with the catalytic cysteine of HMGCS1 following the opening of its lactone ring (33). A subsequent study showed that this HMGCS1-HG adduct is stable *in vitro*, with an estimated half-life of 55 h (28). The same study also showed that HG-mediated inhibition of HMGCS1 in cells is reversed within 30 min after withdrawal when measured by the conversion of radioactive acetate into cholesterol (28). However, the occupancy level of the intracellular HMGCS1 catalytic cysteine and the cause of this increased reversibility of HG remain unclear, primarily due to the lack of tools that allow direct monitoring. Additionally, a comprehensive quantitative analysis of the reactivity of HG toward the global proteome, as well as its effects on reshaping the proteome landscape, has not been reported.

Activity-based protein profiling (ABPP) is a powerful method for assessing the reactivity of small molecules across the global proteome, particularly for small molecules that form covalent bonds with amino acid side chains (34). By directly engaging with the enzyme's active site, ABPP also enables the monitoring of the functional states of target enzyme family members that share similar catalytic mechanisms. Typical activity-based probes (ABPs) consist of a reactive warhead attached to functional groups, such as a fluorescent molecule for visualization or a biotin for streptavidin-bead enrichment and subsequent proteomic analysis. To date, ABPs have been developed for various enzyme families, including kinases, serine proteases, ubiquitin-like activating enzyme families, and many others (35, 36, 37, 38). Among those, iodoacetamide-based ABPPs, which enable the mapping of reactive cysteome under various biological contexts, have become an especially popular tool in discovery science as well as for identifying druggable cysteines (39, 40, 41, 42, 43).

In this study, we developed ABPs for HMGCS1, which are functionalized with either fluorescent dyes or biotin handles. These probes enable the investigation of covalent inhibition effects on HMGCS1 in human cells. By combining these probes with proteomics analyses, we show that HG specifically reacts with the catalytic cysteine of HMGCS1 in cells. HG treatment induces global proteomic changes that are nearly identical to those induced by the HMGCR inhibitor, simvastatin. Our unbiased data support the selective inhibition of mevalonate synthesis by HG and reveal nonredundant functions of HMGCS1 and HMGCR outside the MVP. Consequently, the concurrent targeting of both enzymes potentiates inhibition of mevalonate synthesis, leading to increased anticancer effects in cells and in mouse xenograft models. We also demonstrate that targeted proteolysis of HMGCS1 exhibits a more potent antiproliferation effect than inhibition by HG, due to its poor serum stability. In conclusion, this study presents a comprehensive toolkit for inhibiting and analyzing HMGCS1 activity, providing insights into the cellular and pharmacological effects of HMGCS1 intervention.

## Results

### HG-fluorescence conjugates monitor occupancy of the HMGCS1 active site

**Figure 1:**
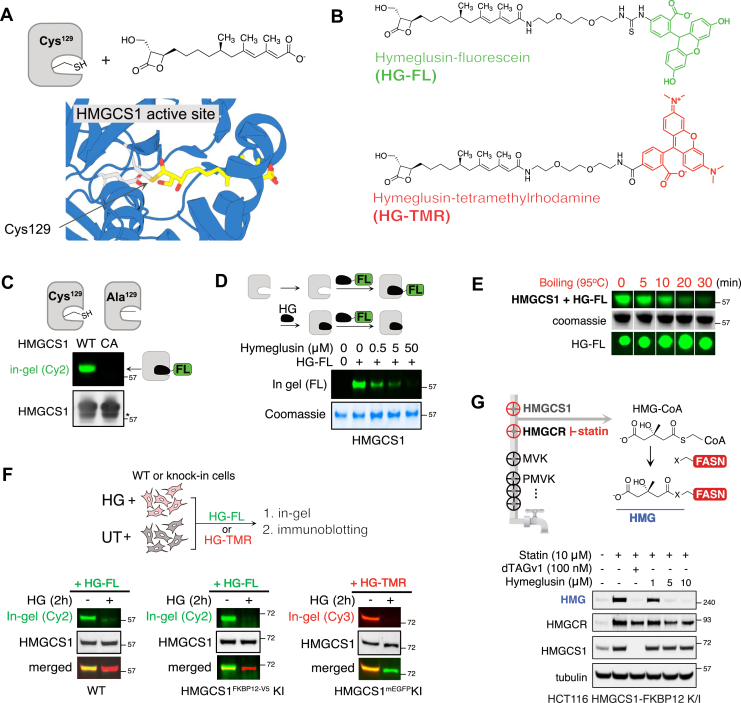
**Hymeglusin-fluorescence conjugates serve as activity-based probes for HMGCS1.***A*, a schematic of the HMGCS1 and Hymeglusin (*top*) and the zoomed-in cocrystal structure of the Hymeglusin-bound HMGCS1 from *Brassica juncea* (*bottom*, PDB: 2F9A). *B*, chemical structures of the Hymeglusin-fluorescein (HG-FL) and Hymeglusin-tetramethylrhodamine probes (HG-TMR). *C*, comparative in-gel fluorescence assay of WT or a catalytically inactive HMGCS1 mutant (C129A, CA) confirms the labeling of the catalytic cysteine of HMGCS1 with HG-FL. *D*, *top:* scheme of the HG-FL competition reaction *in vitro*. *Bottom:* incubating recombinant HMGCS1 (1 μg) with increasing concentrations of Hymeglusin for 30 min results in a decrease of HG-FL–labeled HMGCS1. *E*, the labeling of HMGCS1 by HG-FL was reversed after boiling at 95 °C for at least 20 min in a buffer containing 2% LDS and 50 mM DTT. SDS-PAGE resolved HMGCS1 before the in-gel fluorescence analysis. The stability of the HG-FL probe itself in the given conditions was assessed by dot blot in parallel and showed no change. *F*, HEK293T WT and HCT116 knock-in (KI) (HMGCS1-FKBP12^F36V^ or HMGCS1-mEGFP) cells were treated with Hymeglusin (4 μM for WT, 0.5 μM for KI, 2 h) or left untreated, followed by cell lysis and incubation with HG-FL or HG-TMR for 1 h. After in-gel fluorescence analysis, the gels were transferred to the polyvinylidene fluoride membrane for immunoblotting using an anti-HMGCS1 antibody. *G*, HMGCR inhibition by statins leads to the accumulation of HMG-CoA in cells. This results in the nonenzymatic modification of several residues in the FASN active site by the HMG moiety, which can be detected by the anti-HMG antibody (*top*). HCT116 cells expressing endogenous HMGCS1-FKBP12^F36V^ were treated with the indicated chemicals for 24 h, followed by immunoblotting analysis using anti-HMG, anti-HMGCR, anti-HMGCS1, and anti-tubulin antibodies (*bottom*). FASN, fatty acid synthase; HMGCS1, 3-hydroxy-3-methylglutaryl-coenzyme A synthase 1; LDS, lithium dodecyl sulfate.

HMGCS1 is a 57 kDa protein known to form tight homodimers in cells and *in vitro* (22, 44). A previous cocrystal structure showed that HG forms a covalent bond with the catalytic cysteine of HMGCS1, thereby inhibiting its enzymatic activity (Fig. 1*A*) (45). While HG has been used as a representative inhibitor of HMGCS1 for biological studies (10, 25, 26), its proteome-wide selectivity has not been evaluated quantitatively. This lack of comprehensive evaluation creates uncertainties about its biological implications and on-target effects. We, therefore, synthesized fluorescent probes by conjugating the HG, serving as a warhead, to two different dyes, fluorescein or tetramethylrhodamine (TMR), through a linker (Fig. 1*B*). The Hymeglusin-fluorescein (HG-FL) probe was then incubated with either WT or a catalytically inactive HMGCS1 harboring a Cys-Ala substitution (C129A, CA) *in vitro*, follwing their immunoprecipitation from HEK293T cells ectopically expressing HMGCS1-V5 or HMGCS1 (C129A)-V5 using an anti-V5 nanobody (Fig. 1*C*). In-gel fluorescence analysis showed a signal exclusively in WT HMGCS1, indicating that HG-FL binds the catalytic cysteine residue. Mass spectrometry analysis of recombinant HMGCS1, after incubation with HG, further confirmed the modification of C129 by HG (Fig. S1*A*). When recombinant HMGCS1 was pretreated *in vitro* with different concentrations of HG before adding HG-FL, the fluorescent signal decreased in a dose-dependent manner (Fig. 1*D*). HG-FL binding to HMGCS1 was disrupted in the presence of a reducing agent, dithiothreitol (DTT), which may cleave the thioester bond formed between Cys129 and HG (Fig. 1*E*). However, the strong resistance of this interaction under denaturing conditions implied a tight binding of HG to the hydrophobic pocket, which hinders the access of DTT for *trans-*thiolation reaction. We subsequently investigated whether HG-fluorescence probes could be used to monitor the occupancy of the catalytic cysteine residue within intracellular HMGCS1 (Fig. 1*F*, upper panel). WT HEK293T cells were treated with HG for 2 h, followed by cell lysis, and incubation with the HG probes. Subsequent in-gel fluorescence analyses revealed a fluorescence band that was reversed upon preincubation of cells with HG (Fig. 1*F*, bottom left panel). CRISPR-engineered HCT116 cells expressing endogenous HMGCS1-FKBP12^F36V^ or HMGCS1-mEGFP fusion protein showed similar results when probed by either HG-FL or HG-TMR, with the shifts in molecular weight that match with HMGCS1 chimera (Fig. 1*F*, bottom middle/right panels). The same gels were then subjected to immunoblotting for HMGCS1 detection, and the fluorescent bands coincided with the bands detected by the anti-HMGCS1 antibody. These data indicate that HG-fluorescence probes can serve as ABPs for HMGCS1.

Does HG occupancy of the HMGCS1 catalytic cysteine lead to the depletion of cellular HMG-CoA, the product of HMGCS1 enzymatic activity? Previous investigations have demonstrated that the accumulation of HMG-CoA in cells, resulting from the inhibition of HMGCR by statins, facilitates a nonenzymatic reaction with fatty acid synthase (FASN), leading to the formation of HMG-modified FASN (Fig. 1*G*, upper panel) (46). The presence of this modification can be effectively monitored using an anti-HMG antibody (47). Notably, prior studies have shown that the overall reactivity of this antibody correlates with intracellular levels of HMG-CoA. Indeed, when HCT116 cells expressing endogenous HMGCS1-FKBP12^F36V^ fusion protein were treated with simvastatin, an HMG positive band appeared around 240 kDa range (Fig. 1*G*, bottom panel, first two lanes). The addition of the dTAGv1 ligand, which recruits the von Hippel-Lindau (VHL)-containing E3 ligase complex to the FKBP12^F36V^ tag and induces the degradation of HMGCS1-FKBP12^F36V^chimera, caused the disappearance of the HMGylated protein signal (Fig. 1*G*, bottom panel, third lane, and Fig. S1, *B* and *C*) (22, 48). This indicates that the cellular HMG-CoA level dropped in the absence of HMGCS1, serving as the positive control for this assay system. Treatment with 5 μM concentrations of HG similarly led to a significant decrease in the HMGylated protein intensity, indicating inhibition of HMGCS1 activity (Figs. 1*G* and S1, *C*–*E*). Of note, statin treatment induced a substantial increase in HMGCS1 and HMGCR levels due to the feedback effect mediated by the sterol regulatory element-binding protein 2 (SREBP2) transcription factor (49).

### HG reacts highly selectively with HMGCS1 in human cells

**Figure 2:**
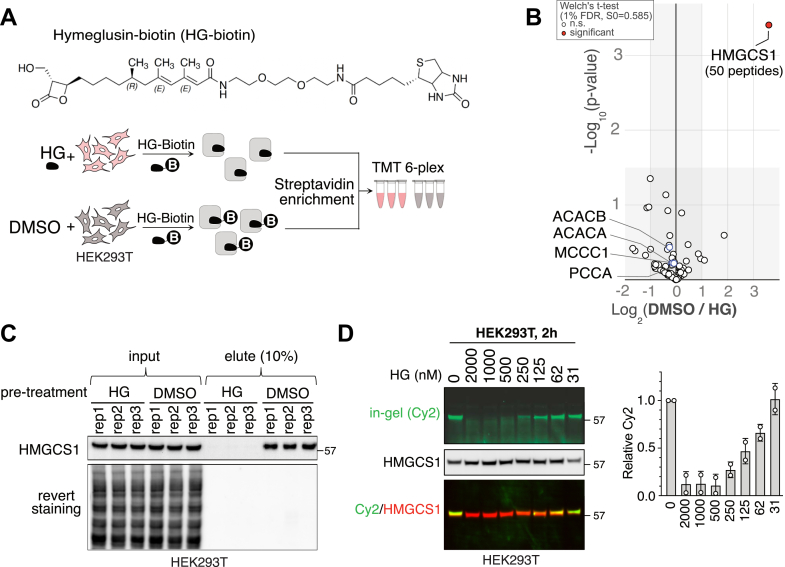
**Hymeglusin is a highly selective inhibitor of HMGCS1 in human cells.***A*, the structure of the Hymeglusin-biotin probe is shown at the *top*, and the workflow of competitive affinity purification mass spectrometry (AP-MS) using Hymeglusin-biotin combined with TMT-6 plex methods is shown at the *bottom*. *B*, HEK293T cells were either treated with HG (1 μM, 30 min) or dimethyl sulfoxide, followed by further incubation with the in-house HG-biotin probe (1 μM, 2 h). After streptavidin beads enrichment, the eluates were analyzed through tandem-mass-tag–based proteomic analysis. The proteomic data are presented as a volcano plot of the –log_10_-transformed *p* value *versus* the log_2_-transformed ratio of dimethyl sulfoxide/Hymeglusin pre-treated cells. n = 3 biological replicates. *p*-values were calculated by two-sided Welch’s *t* test (adjusted to 1% FDR for multiple comparisons, S0 = 0.585). *C*, HEK293T cells were treated as described in *panel A*. After streptavidin enrichment, the eluates were subjected to immunoblotting using the anti-HMGCS1 antibody. Rep: replicate. *D*, HEK293T cells were treated with increasing concentrations of Hymeglusin for 2 h, followed by HG-FL treatment and in-gel fluorescence analysis. The same extracts were then transferred to a polyvinylidene fluoride membrane to probe for HMGCS1 using immunoblotting. The average of two replicates is shown on the *right*. FDR, false discovery rate; HG, Hymeglusin; HG-FL, Hymeglusin-fluorescein; HMGCS1, 3-hydroxy-3-methylglutaryl-coenzyme A synthase 1; TMT, tandem mass tag.

We next evaluated the reactivity of HG toward the human proteome using an unbiased chemical proteomics approach. To identify the HG-labeled proteins using mass spectrometry, we synthesized the HG-biotin probe (Fig. 2*A*, top). HEK293T cells were either treated with HG or dimethyl sulfoxide (DMSO) for 30 min prior to incubation with HG-biotin. The proteins labeled with HG-biotin were enriched using streptavidin beads, and the eluates were analyzed using a 6-plex tandem mass tag (TMT)-based quantification. Our proteomic analysis revealed that HMGCS1 is the only significantly enriched protein that was displaced by the pretreatment with HG (12-fold increase, 50 peptides detected), demonstrating HG’s high selectivity for HMGCS1 (Fig. 2*B*). An orthogonal immunoblotting assay confirmed the strong enrichment of HMGCS1 by HG-biotin in DMSO pre-treated cells (Fig. 2*C*). In contrast, endogenously biotinylated proteins such as acetyl-CoA carboxylase 1 (18 peptides), propionyl-CoA carboxylase (19 peptides), and methylcrotonyl-CoA carboxylase (14 peptides) were enriched regardless of HG pretreatment, as a consequence of their interaction with streptavidin (Table S1). Consistently, the HG-FL assay on HEK293T cells that were pretreated with increasing concentrations of HG revealed a single strong fluorescence signal around 57 kDa, corresponding to the molecular weight of HMGCS1 (Fig. 2*D*). This signal disappeared when the cells were pretreated with over 250 nM HG. We conclude that HG is a potent and selective inhibitor of HMGCS1, making it a suitable tool for examining the downstream biological effects of acute HMGCS1 inhibition.

### The inhibition or degradation of HMGCS1, or inhibition of HMGCR, results in identical changes to the proteome

**Figure 3:**
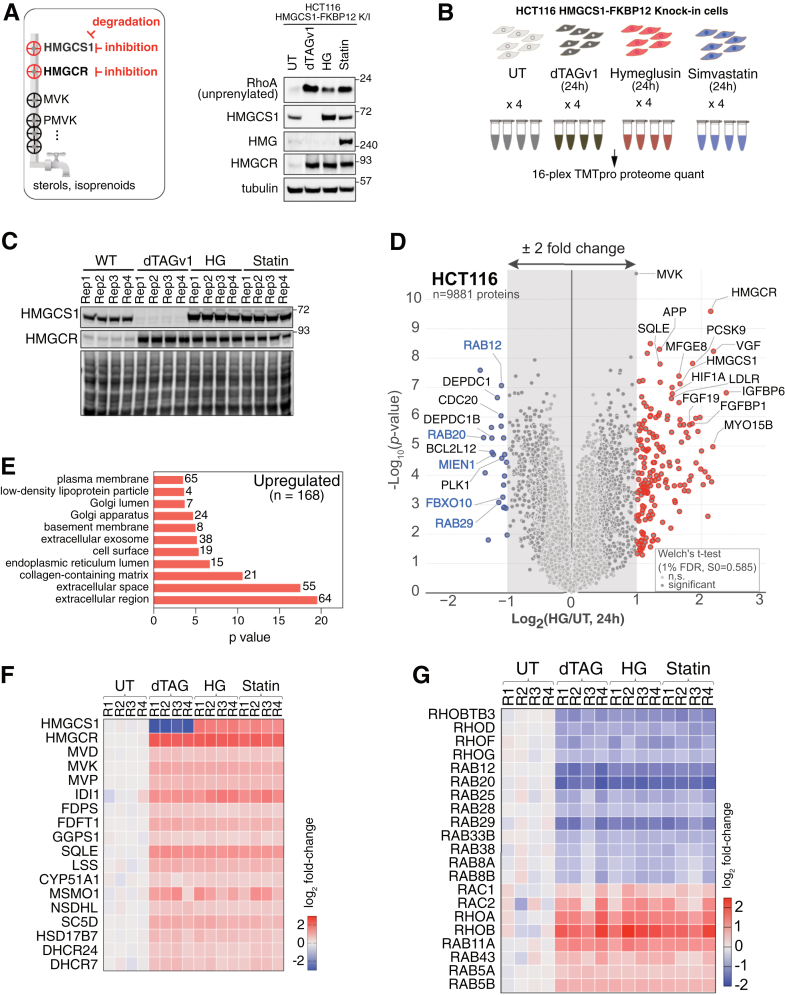
**Perturbing HMGCS1 by hymeglusin and HMGCR by simvastatin leads to identical global proteome changes.***A*, *left panel:* a schematic illustrating the mevalonate pathway flux. Three perturbation approaches—inhibition or degradation of HMGCS1 and inhibition of HMGCR—are compared for their effects on global proteome changes. *Right panel:* HCT116 HMGCS1-FKBP12^F36V^ knock-in cells were treated with dTAGv1 (100 nM, for HMGCS1 degradation), Hymeglusin (5 μM, for HMGCS1 inhibition), and simvastatin (10 μM, for HMGCR inhibition) for 48 h. The cell extracts were then probed with the indicated antibodies to check the cellular response. *B*, the 16-plex TMTpro approach was utilized to compare the global proteome changes after 24 h of the specified small molecule treatment. *C*, HEK293T cells treated as described in *panel B* were probed with the indicated antibodies as a quality control step prior to proteomic analysis. *D*, analysis of the TMTpro-plex data is presented as a volcano plot of the −log_10_-transformed *p* value *versus* the log_2_-transformed ratio of Hymeglusin-treated/untreated (UT) conditions for HCT116 HMGCS1-FKBP12^F36V^ knock-in cells. *p* values were calculated by two-sided Welch’s *t* test (adjusted to 1% FDR for multiple comparisons, S0 = 0.585). Of the statistically significant hits, proteins with more than a 2-fold increase are *circled in red* (168 proteins), while those with more than a 2-fold decrease are in *blue*. A total of 9881 proteins were quantified. n = 4 biological replicates. *E*, gene ontology analyses of the significantly upregulated proteins with more than 2-fold changes, 168 proteins as described in *panel D*, are shown. N represents the number of proteins counted in the category. *F*, 19 quantified proteins in the mevalonate/sterol pathway were curated, and their log2 fold changes are presented as a heat map. dTAG treatment led to the steep depletion of HMGCS1. *G*, representative families of isoprenylation targets (Rho, Rab, Rheb, and Ras proteins) were curated from 1200 statistically significant hits and plotted as a heat map. FDR, false discovery rate; HMGCR, HMG-CoA reductase; HMGCS1, 3-hydroxy-3-methylglutaryl-coenzyme A synthase 1.

To gain a deeper understanding of the cellular response to the inhibition of HMGCS1, we analyzed global proteomic changes following HG treatment (Fig. 3*A*, left). We compared the proteome alterations caused by the acute degradation of HMGCS1 by using the HCT116 HMGCS1-FKBP12^F36V^ knock-in (KI) cells, which may decouple any side effects of HG and nonenzymatic roles of HMGCS1, if they exist. We also compared the proteome changes induced by the inhibition of HMGCR, as HMGCS1 and HMGCR are the first two enzymes in the MVP. If these two enzymes have additional functions outside the MVP, it may be evident in the proteome changes. Immunoblotting analysis showed that dTAGv1 treatment for 24 h led to the expected depletion of HMGCS1 and the upregulation of unprenylated RhoA and HMGCR, a known feedback effect (Fig. 3*A*, right) (50). HG treatment also resulted in an increase of RhoA and HMGCR, along with HMGCS1. Similarly, statin treatment caused a significant increase in RhoA, HMGCR, and the emergence of HMGylated FASN. These data suggest that each condition results in a comparable degree of mevalonate flux perturbation. Accordingly, the TMT-based proteomics approach was applied to the cells under the corresponding conditions in four replicates, and the overall quality of the samples was assessed again through immunoblotting (Fig. 3, *B* and *C*). The following mass spectrometry analysis revealed the relative abundance of 9881 proteins across all 16 samples without missing values (Table S2). Principal component analysis showed that replicates of each sample clustered together, indicating consistency among the replicates (Fig. S2*A*). Notably, only the untreated cells showed a distinct separation, while the cells treated with the MVP inhibition formed a closely clustered group. For example, proteome profiles from cells treated with simvastatin and HG showed nearly identical results, indicated by a strong Pearson correlation coefficient of 0.97 (Fig. S2*A*). A similarly high correlation coefficient (Pearson r = 0.95) was observed when comparing HG-treated cells with those subjected to HMGCS1 degradation (Fig. S2*C*). These unbiased proteomics data reveal that 1) HG produces proteome changes that are highly specific to HMGCS1 inhibition, 2) HMGCS1 depletion causes proteome changes driven by the loss of its enzymatic function, and 3) targeting HMGCS1 and HMGCR leads to nearly identical proteomic changes in 24 h, reinforcing their essential role in the MVP.

Twelve percent of the total proteome (1200 of 9881 proteins) showed significant changes within 24 h of HG treatment. It was evident that the inhibition of the MVP substantially stimulated the production of a subset of the proteome, with 168 proteins increasing by more than 2-fold while 24 proteins decreased (Fig. 3*D*). Gene ontology (GO) analysis of the significantly upregulated proteins (>2-fold, 168 proteins) for their cellular compartment indicated that the proteins in the secretory pathway are significantly enriched (Fig. 3*E*). Functionally, proteins involved in regulating cell migration or adhesion and signaling receptor binding were prominent (26 and 27 proteins, respectively), aligning with the role of the MVP in regulating small GTPase family members, such as Rho, Rab, and Ras. A notable increase was also observed in ∼20 enzymes within the MVP across the various treatment conditions (Fig. 3*F*). All three inhibitory conditions resulted in a similar induction of the MVP enzymes. These findings are consistent with prior reports of a transcriptional feedback mechanism mediated by SREBP2, which is triggered upon the inhibition of MVP enzymes (51). Significantly downregulated proteins (greater than 2-fold difference, 24 proteins) included seven proteins involved in the mitotic spindle checkpoint, indicating that cell cycle arrest was initiated, likely as a sign of early-stage apoptosis (Fig. 3*D*). In this context, BCL2L12, an antiapoptotic regulator, was downregulated by more than 2-fold. Mevalonate is a precursor for not only sterols but also isoprenoids that are essential for posttranslational modifications, such as farnesylation and geranylgeranylation. Three proteins targeted by farnesylation (RAB20, RAB29, and RAB12) and two proteins for geranylgeranylation with a CAAX motif (MIEN1 and FBXO10) were significantly downregulated, suggesting their destabilization due to the loss of prenylation. When we examined the changes in prenylation substrates on a broader scale, we found many of them among the statistically significant hits. Intriguingly, they showed divergent expression changes upon the inhibition of mevalonate production (Fig. 3*G*). Their downregulation may stem from instability caused by the lack of prenylation in the newly produced proteins, while the upregulated proteins may be a result of their transcriptional feedback effect mediated by SREBP2, producing unprenylated Rho and Rab proteins. In any case, their cellular functions may be negatively affected by the loss of prenylation. In summary, the inhibition or degradation of HMGCS1, or the inhibition of HMGCR by statins, results in a similar alteration of the global proteome that is characterized by the induction of the MVP enzymes and the protein substrates of the downstream metabolites, isoprenoids.

### Simultaneous targeting of HMGCS1 and HMGCR produces a synergistic anticancer response

Repurposing statins has attracted significant interest as a strategy for treating cancer (20). However, many patients were nonresponsive to statin treatment. Prior studies have suggested that cells demonstrating feedback upregulation of the MVP enzymes, particularly HMGCS1, tend to exhibit strong statin resistance (52, 53, 54). We tested this by using seven cell lines with different lineages. Simvastatin treatment for 24 h led to varying levels of HMGCS1 induction (Fig. S3, *A* and *B*). Among the seven cell lines, HCT116 showed a 2.5-fold increase in HMGCS1, while the HMGCS1 levels in HEK293T or MFE296 cell lines remained unaltered (Fig. S3*C*). The following cell viability assay on HCT116 and HEK293T cells indicated that HCT116 cells are highly resistant to simvastatin treatment, while HEK293T cells were relatively more sensitive (Fig. S3*D*). Colony formation assays on HCT116 and MFE296 cells also showed resistance for HCT116, whereas MFE296 cells exhibited a more sensitive response (Fig. S3*E*). Based on our data and the prior study, we conclude that the increased production of HMGCS1 upon statin treatment may contribute to the low efficacy of statins in HCT116.

**Figure 4:**
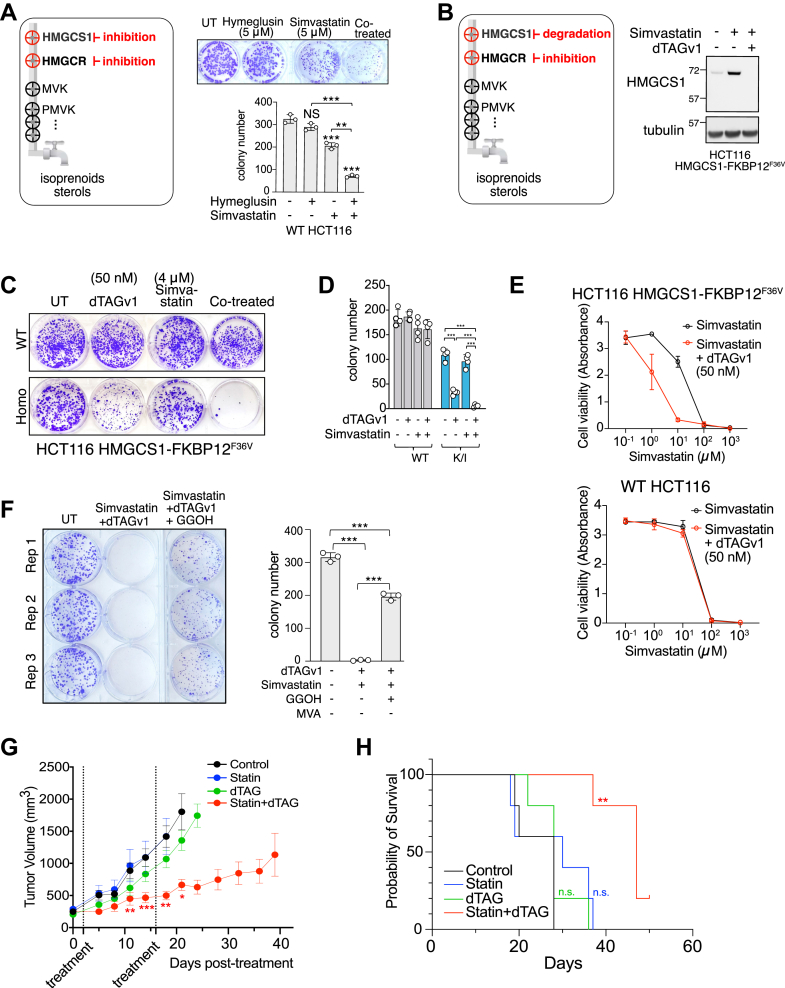
**Combinatory targeting HMGCS1 and HMGCR exhibit synergistic antitumor effects.***A*, the *left panel* presents a schematic illustration of the combinatorial inhibition of HMGCS1 and HMGCR. In the *right panel*, the colony formation assay results for HMGCS1-FKBP12^F36V^ K/I HCT116 cells are shown, where treatments involved either Hymeglusin (5 μM), simvastatin (5 μM), or a combination of both inhibitors. Corresponding inhibitors were applied on days 5 and 7 after seeding, and the colony numbers were counted on day 12. The accompanying quantification graph displays the means ± SD from biological triplicates. *B*, the *left panel* illustrates the degradation of HMGCS1 and the inhibition of HMGCR as a combinatorial approach to target the mevalonate pathway. The *right panel* shows the induction of HMGCS1 following simvastatin treatment, which was completely reversed by dTAGv1 treatment due to the induced degradation in HCT116 cells expressing endogenous HMGCS1-FKBP12^F36V^. *C*, HMGCS1 degradation by dTAGv1 (50 nM) increases the inhibitory effects of simvastatin (4 μM) on the colony-forming activity of HCT116 HMGCS1-FKBP12^F36V^ cells. *D*, means ± SD of biological quadruplicate data from *panel C*. *E*, HMGCS1 degradation by the dTAG system reduces the IC_50_ value of simvastatin by 10-fold in HCT116 HMGCS1-FKBP12^F36V^ knock-in cells (*top*). Cell viability was assessed 24 h after treating the corresponding cells with increasing concentrations of simvastatin and dTAGv1 (50 nM). The WT HCT116 did not show the synergistic effect of dTAGv1 and simvastatin (*bottom*). Means ± SD of biological quadruplicate. *F*, the anticolony formation effect induced by the HMGCS1 degradation and HMGCR inhibition was rescued by supplementation of geranylgeraniol (GGOH, 40 μM). HCT116 HMGCS1-FKBP12^F36V^ K/I cells were treated with dTAGv1 (50 nM) and simvastatin (4 μM) on days 5 and 7, postseeding. GGOH (40 μM) was added to the media of the corresponding cells on days 5 and 7. Means ± SD of biological triplicate data are shown on the *right*. *G*, HMGCS1-FKBP12^F36V^ K/I HCT116 cells were implanted into nude mice. When the tumor size reached 200 to 250 mm^3^, we administered two treatment regimens (shown in *dotted vertical lines*), including saline control, 5 mg/kg simvastatin *via* gavage three times a week, weekly 5 mg/kg dTAGv1 through i.p., and the combination of both dTAGv1 and simvastatin. Degradation of HMGCS1 by the dTAG system potentiates the tumor growth-suppressing effect of simvastatin. Means ± SEM of five mice per condition are plotted. A paired *t* test between control and statin + dTAG treatment was performed for days 11, 14, 18, and 21. ∗*p* < 0.05, ∗∗*p* < 0.01, ∗∗∗*p* < 0.001, and ∗∗∗∗*p* < 0.0001. *H*, survival probability analysis of the mice presented in *panel H* using Kaplan–Meier software reveals extended mortality in the dTAG and statin cotreatment groups compared to the other control groups. Log-rank (Mantel-Cox) test was performed between the control and statin + dTAG treatment groups. ∗∗*p* < 0.01. HMGCR, HMG-CoA reductase; HMGCS1, 3-hydroxy-3-methylglutaryl-coenzyme A synthase 1.

The strong correlation observed between changes in the global proteome caused by the inhibition of HMGCS1 and HMGCR suggests that simultaneously targeting both enzymes may more effectively inhibit the mevalonate metabolic pathway. This could address the current limitation of statin efficacy in anticancer therapy, especially in cells that are resistant to statins. We, therefore, examined if simultaneous targeting of HMGCS1 and HMGCR would enhance the antiproliferative effect of the monotherapy using HCT116 cells (Fig. 4*A*). In a colony formation assay, treatment with a statin alone resulted in a ∼30% decrease in the number of colonies, while HG alone had a negligible effect. In contrast, combined treatment with simvastatin and HG resulted in significant inhibition of colony formation. Similarly, we examined the effect of HMGCS1 degradation on cell proliferation, both individually and in combination with a statin, using HCT116 cells expressing endogenous HMGCS1-FKBP12^F36V^ fusion protein (Fig. 4*B*). In these cells, simvastatin treatment resulted in a substantial increase in endogenous HMGCS1-FKBP12^F36V^, as expected, whereas addition of dTAGv1 nearly depleted HMGCS1 *via* induced degradation. Treatment of dTAGv1 in these cells led to a 60% reduction in colony numbers, while cotreatment with dTAGv1 and simvastatin resulted in nearly complete inhibition of colony formation (Fig. 4, *C* and *D*). Similarly, cell viability assay results gained from the monolayered cell culture system indicated that the addition of dTAGv1 ligand significantly enhanced the IC_50_ of simvastatin, decreasing from 15.6 μM to 1.3 μM (Fig. 4*E*). As a control, we treated WT HCT116 cells with the dTAGv1 ligand, which exhibited no change in the IC_50_ of simvastatin, confirming that the observed change in IC_50_ was due to the degradation of HMGCS1 (Fig. 4*E*, bottom). Importantly, the cell death caused by the joint treatment was rescued by supplementing the media with geranylgeranyl alcohol, a precursor of the downstream metabolite of mevalonate, indicating that the enhanced cytotoxicity arises from the impairment of the mevalonate synthesis (Fig. 4*F*).

Next, we performed the equivalent study using the mouse xenograft model after implanting HCT116 HMGCS1-FKBP12^F36V^ cells (Fig. 4, *G* and *H*). Four groups of mice were subjected to two treatment regimens over a 2-week interval, including saline control, oral simvastatin three times a week, weekly i.p. injection of dTAGv1, and the combination of simvastatin and dTAGv1. The group of mice that received both simvastatin and dTAGv1 exhibited a synergistic effect on tumor growth suppression that lasted for 40 days. In contrast, administration of simvastatin or dTAGv1 ligand injection alone did not show a significant difference compared to the PBS-treated control group, highlighting the synergistic effect of HMGCS1 degradation and HMGCR inhibition in the xenograft model.

Finally, we investigated the impact of HMGCS1 degradation in cells strongly resistant to statins. Since statin resistance has been previously identified as a factor contributing to the failure of statins in cancer therapy, it would be clinically valuable if modulating HMGCS1 could enhance the effectiveness of statins through a synergistic effect. HCT116 cells were treated with increasing doses of statin (starting from 5 to 30 μM) for 4 months, after which the IC_50_ of these cells became 6.3-fold higher than that of the parental HCT116 cells (Fig. S4*A*). After testing the expected loss of HMGCS1 upon dTAGv1 treatment or an increase of HMGylated protein levels upon inhibition of HMGCR (Fig. S4*B*), the effect of HMGCS1 degradation alone (Fig. S4*C*) or in combination with simvastatin treatment was examined in these cells (Fig. S4, *C* and *D*). Overall data suggest that dual targeting of HMGCS1 and HMGCR may offer potential benefits in both statin-sensitive and resistant cell types, and the targeted proteolysis approach for HMGCS1 perturbation could demonstrate greater efficacy than inhibition.

### HG warhead exhibits poor serum stability

**Figure 5:**
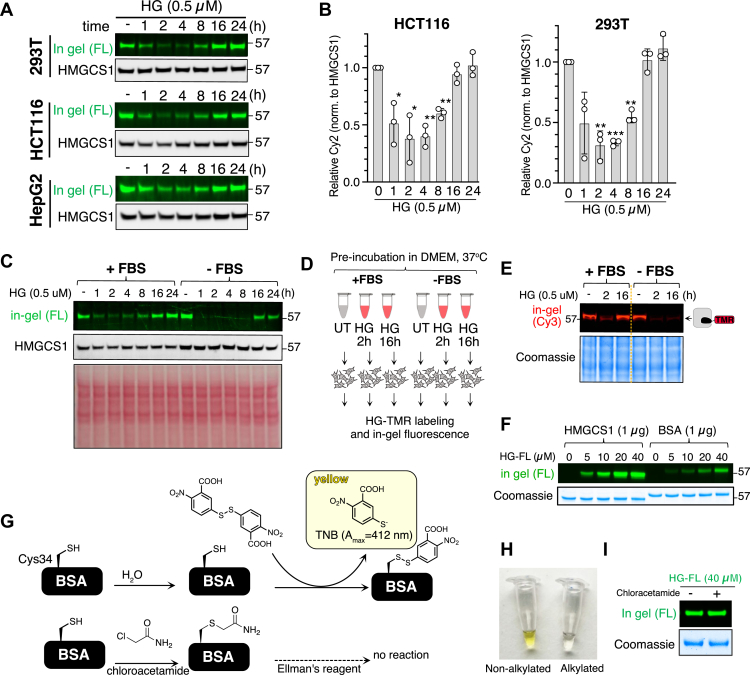
**Hymeglusin exhibits poor serum stability.***A*, HCT116, HEK293T, and HepG2 cells were treated with Hymeglusin (0.5 μM) for the indicated time points, followed by lysis and reaction with the HG-FL probe. In-gel fluorescence analysis and immunoblotting were then performed (*B*) The quantification of relative fluorescence intensity from three replicate experiments in *panel A* is presented. Mean ± SD. *C*, the absence of fetal bovine serum (FBS) in the cell culture media delays the appearance of active HMGCS1 with free catalytic cysteine after prolonged treatment of cells with Hymeglusin. *D*, a workflow to test the effect of FBS on the stability of Hymeglusin. A total of 0.5 μM of Hymeglusin was added to DMEM supplemented with or without 10% FBS. The media were then incubated at 37 °C for 2 or 16 h before being added to HEK293T cells. *E*, HEK293T cells treated as in *panel D* were subjected to in-gel fluorescence analysis. *F*, *in vitro* incubation of HG-FL with recombinant HMGCS1 (1 μg) or BSA (1 μg) for 1 h produces a green fluorescence signal in the relevant molecular weight regions, indicating a strong interaction between HG-FL and BSA. *G*, scheme of Ellman’s reagent, which turns the solution into a yellow visible color when reacted with thiol by producing TNB. *H*, a portion of the samples treated as in (*G*) were reacted with Ellman’s reagent. Specifically, BSA was incubated with water or chloroacetamide for 10 min, followed by the addition of Ellman’s reagent. The water pretreated sample turned into the expected yellow solution, while the chloroacetamide pretreated BSA solution did not change color, indicating the loss of free thiol due to alkylation. *I*, the remaining portion of the samples in *panels G and H* was then incubated with HG-FL, which showed an equal level of *green* fluorescence signal. BSA, bovine serum albumin; DMEM, Dulbecco’s modified Eagle’s medium; HG-FL, Hymeglusin-fluorescein; HMGCS1, 3-hydroxy-3-methylglutaryl-coenzyme A synthase 1; TNB, 2-nitro-5-thiobenzoate.

Despite identical proteome changes upon degradation or inhibition of HMGCS1 (Fig. 3), enzymatic inhibition of HMGCS1 by HG did not elicit any cytotoxic effects in either HCT116 or HEK293T cells when used alone (Figs. 4*A* and S4*A*). This observation is inconsistent with the antitumor efficacy of dTAGv1-mediated HMGCS1 degradation, prompting us to further investigate the long-term stability of HG. Prior investigations using murine models administered with HG reported an 80% reduction in cholesterol synthesis within 30 min and a complete loss of inhibitory function by 180 min when measured by the conversion of ^14^C-labeled acetate into plasma cholesterol (27, 28). It was speculated that this effect may be due to the reversible nature of the thioester bond formed between HG and HMGCS1. However, the precise kinetics underlying the decline in HG efficacy and the mechanism behind its loss of efficacy in cells remain to be determined. Considering that researchers have utilized HG as an inhibitor of HMGCS1 in a range of cell culture models, elucidating this limitation could be crucial for its appropriate application. Our use of the HMGCS1 ABPs enabled us to monitor the occupancy of the catalytic cysteine in HMGCS1 by HG over time. After 8 h of treatment with 0.5 μM HG, we observed the emergence of the catalytically active HMGCS1 that was labeled with HG-FL, a trend that became more pronounced after 16 h in HEK293T, HCT116, or HepG2 cells (Fig. 5, *A* and *B*). These data suggest that the inhibition of HMGCS1 by HG does not persist over the long-term incubation, likely due to either the reversal of the thioester bond between HMGCS1 and HG or the newly synthesized HMGCS1 not being charged by HG. This activity profiling data aligns with the absence of sustained efficacy in the antiproliferative effects we observed with HG treatment. Interestingly, the removal of serum from the culture media led to a delayed restoration of active HMGCS1 levels (Fig. 5*C*). To test whether this observation was due to the direct impact of serum on HG stability, we preincubated HG in Dulbecco’s modified Eagle’s medium (DMEM) with 10% fetal bovine serum (FBS) for 16 h before being added to the cells and found that the catalytic cysteine of HMGCS1 was not effectively inhibited anymore (Fig. 5, *D* and *E*). In contrast, preincubation in DMEM without 10% FBS did not reduce the efficacy of HG. Additionally, we observed intense labeling of bovine serum albumin (BSA) by HG-FL after incubating them for 1 h *in vitro* (Fig. 5*F*). The labeling persisted even after boiling for 5 min in denaturing conditions, suggesting a strong interaction between HG-FL and BSA. We tried to clarify this by categorizing the potential pathways that underlie how BSA impacts the efficacy of HG into two distinct types: first, BSA contains 35 cysteines, of which 34 form disulfide bonds, leaving one reactive cysteine (55). Thus, HG may form a covalent bond with BSA *via* reactive cysteines, including Cys34. The second possibility is that HG forms a noncovalent, yet strong, interaction through the fatty acid binding sites, as BSA has several fatty acid binding sites and HG resembles the structure of fatty acids (56). As the second possibility is rather complex to address, we evaluated the first hypothesis. If the cysteine on the BSA is reacting with HG-FL, then alkylating BSA with chloroacetamide prior to HG-FL incubation should result in no labeling. We thus treated BSA with chloroacetamide, and the loss of reactive cysteine was confirmed by using Ellman’s reagent (Fig. 5, *G* and *H*). When native BSA was incubated with Ellman’s reagent, the solution changed to yellow due to the formation of 2-nitro-5-thiobenzoate. After preincubating the BSA with chloroacetamide, we observed the expected loss of color change, representing the absence of reactive cysteine on BSA (Fig. 5*H*). Then, this alkylated BSA was treated with HG-FL, with nonalkylated BSA serving as a positive control (Fig. 5*I*). The subsequent in-gel fluorescence analysis revealed an unchanged fluorescence signal, suggesting that HG likely binds to BSA through non-cysteine–mediated interactions. Together, poor serum stability may limit the long-term use of HG in cell and *in vivo* studies, as human serum typically contains ∼40 mg/ml of albumin.

## Discussion

The MVP is a well-established focus of interest for chemists and biologists, given its critical role in cholesterol biogenesis and prenylation. Prenylation, in particular, is essential for the proper function of several oncogenic proteins, such as Ras, which underscores its contribution to cancer progression. Consequently, several enzymes in the MVP have received substantial attention in pharmaceutical research for inhibitor development. However, the initial enzyme of the pathway, HMGCS1, has received relatively little focus, overshadowed by HMGCR, and the established use of statins. We have recently discovered that HMGCS1 functions as a gatekeeper of this pathway, especially in cells with hyperactive mTORC1 signaling (22). HMGCS1 senses and responds to fluctuations in mTORC1 activity, paralleling the critical role of HMGCR in sensing and responding to changes in membrane sterol levels. Moreover, the presence of a ligandable cysteine in HMGCS1 makes it a particularly unique and advantageous target for probe development, which can significantly facilitate investigations into both the mechanisms of inhibitor action and potential resistance.

Currently, few tools are available to interrogate HMGCS1. Among them, HG, a natural product derived from fungi, stands out as the primary tool utilized in the field since its discovery in the 1980s (27, 28, 29, 30, 31, 57). Despite its widespread use, the selectivity and overall efficacy of HG in cells have not been thoroughly evaluated since the 1990s, raising concerns about its suitability as a tool compound. This prompted us to assess its cellular potency and selectivity by developing ABPs inspired by the HG warhead and leveraging modern technologies, such as proteomics. Our unbiased analyses, grounded in chemical biology approaches, revealed that HG is a potent and highly selective inhibitor of HMGCS1 in HEK293T cells, with a concentration of 0.5 μM fully saturating the catalytic cysteine of HMGCS1 in cells. However, we also determined that HG has extremely poor serum stability, apparently derived from the interaction with serum albumin, which is comparable to on-target binding to HMGCS1. This drawback limits the duration and efficacy of HG to target HMGCS1. Nevertheless, our comprehensive data led us to consider HG a viable tool for studying the short-term pharmacologic effects of HMGCS1 inhibition, provided we carefully monitor its intracellular stability and activity with our ABPs.

After carefully characterizing HG, we explored the effect of inhibiting HMGCS1 using HG and an additional method that incorporates a chemical genomic approach to induce the degradation of HMGCS1. Proteomic analysis of cells following HMGCS1 inhibition or degradation, or HMGCR inhibition, revealed surprisingly similar proteome changes in 24 h. This result indicates that targeting both enzymes simultaneously may enhance drug efficacy without amplifying the risk of potential side effects from either inhibitor. Our follow-up cell biological and xenograft studies support this notion that dual targeting of HMGCS1 and HMGCR potentiates the anticancer effect of statin monotherapy. Overall, our findings highlight the potential of targeting HMGCS1 either as a monotherapy or in combination with statins, emphasizing the benefits of the induced proteolysis approach in this context.

Comparative analysis of HMGCS1 inhibition by HG or of HMGCS1 degradation using the chemically induced tag system (dTAG) demonstrated the superior potency of the degradation approach in inhibiting cancer cell proliferation. Heterobifunctional degrader molecules have potential therapeutic advantages over the monofunctional inhibitor (58, 59). In the case of HMGCS1, the proteolysis targeting chimera (PROTAC) degrader can counteract the compensatory upregulation of HMGCS1 through its degradation. As the half-life of the HMGCS1 protein spans several days, the impact of drug-induced degradation of HMGCS1 can be prolonged longer than the inhibitors. Additionally, covalent PROTACs have been shown to enhance cellular uptake and target selectivity (60). Therefore, developing small molecules that could induce targeted proteolysis of HMGCS1 may offer better pharmacological properties than inhibitors, providing a more effective strategy to treat cancer both as monotherapy and in combination with HMGCR inhibitors. In this context, we investigated whether we could convert the HMGCS1 inhibitor into a bifunctional molecule by conjugating HG to a ligand that recruits either cereblon or VHL E3 ligase complex with varying linkers (Fig. S6*A*). Comparative degradation analysis of the five HG-PROTAC molecules in HEK293T, RPE1, and HeLa cells consistently showed that HG-PROTAC4, which recruits the VHL E3 complex and contains three PEG in the linker, induces the most pronounced reduction of HMGCS1, in the presence of translation inhibitor cycloheximide (Fig. S6*B*). The reduced HMGCS1 level observed with HG-PROTAC4 treatment was reversed in the presence of the ubiquitin-activating E1 enzyme inhibitor, MLN7243 (Fig. S6*C*). In-gel fluorescence analysis of HG-PROTAC4–treated cells using HG-FL showed that HG-PROTAC4 enters cells within 15 min, despite its large molecular weight of 924 Da, and that it fully occupies the catalytic cysteine of HMGCS1 (Fig. S6*D*). However, HMGCS1 degradation occurs at a slower rate, with a 50% reduction observed after 8 h. These data indicate that HG-PROTAC4 initially functions as an inhibitor, as early as 15 min, and subsequently as a degrader *in situ*. Our findings establish a rationale for the development of more efficacious bifunctional degraders that target the HMGCS1 catalytic cysteine.

Although we here defined that HG still interacts with albumin after alkylating all the reactive cysteines on them, further investigation is needed to determine the detailed binding mode between HG and albumin protein (61). One possibility is that the structure of HG resembles that of fatty acids. Albumin has well-characterized fatty acid binding pockets, with at least seven known pockets that could potentially interact with HG (62). Another limitation of the present study is that the mitochondrial HMG-CoA synthase, HMGCS2, which shares approximately 67% sequence identity with HMGCS1, may be another potential target of HG. HMGCS2 shows tissue-specific expression, with high levels in hepatocytes, unlike the universal expression of HMGCS1. Our competitive affinity purification followed by immunoblotting shows that the HG-biotin probe does not pull down HMGCS2, in contrast to its effective isolation of HMGCS1 in a hepatocyte cell line, HepG2, under the current experimental conditions (Fig. S6, *E* and *F*). Nonetheless, our data do not fully rule out the possibility that HG may target HMGCS2 in certain biological contexts.

In summary, this study reports new chemical biology tools for an important yet underexplored metabolic enzyme within cells, HMGCS1. It offers insights into how targeting HMGCS1—either alone or together with HMGCR—can lead to alterations in the identical cellular proteome while potentiating their antiproliferation effect. Furthermore, the study holds potential for developing drug-like inhibitors or bisubstrate degraders of HMGCS1 by utilizing the ABPs developed in this study.

## Experimental procedures

Detailed information about reagents and resources used in this study is displayed in Table S3.

### Cell culture

HEK293T, HCT116, RPE1, HeLa, MCF7, HepG2, U2OS, DLD1, and MFE296 cells were grown in DMEM, supplemented with 10% FBS and maintained in a 5% CO_2_ incubator at 37 °C.

### Generation of KI cell lines using CRISPR-Cas9 gene editing

HMGCS1-FKBP12^F36V^-V5 KI HCT116 and HMGCS1-mEGFP KI HCT116 cells were generated based on our previous paper (22). Briefly, HCT116 cells were transfected with gRNA (5′-GAACATTAAGATACTCTGTG-3′) assembled into the pX459 vector and donor vector containing FKBP12^F36V^ or mEGFP. After antibiotic selection, the cells were separated into single clones by serial dilution (for FKBP12^F36V^) or by fluorescence-activated cell sorting (for EGFP). Three weeks later, the single clones were expanded and screened by immunoblotting using an anti-HMGCS1 antibody to confirm transgene integration.

### Generation of stable cell lines

Stable cell lines were generated according to the previously reported method (22). HEK293T cells were transfected with pHAGE-HMGCS1-V5 or pHAGE-HMGCS1 (C129A)-V5. Virus-containing media were collected 24 h and 48 h after transfection and filtered with 0.45 mm filters. HEK293T cells were infected with the viral solution, and puromycin selection was performed.

### Cell lysis and immunoblotting assay

Cells were plated 24 h before being treated with inhibitors, and cell confluency did not exceed 70% at the time of harvest. Cell pellets were lysed with either in-house radioimmunoprecipitation assay buffer (RIPA) buffer (50 mM Hepes, 150 mM NaCl, pH 7.6, 1% NP-40, 1% sodium deoxycholate, 0.1% SDS, 10 mM glycerophosphate, 10 mM sodium pyrophosphate, protease inhibitor cocktail, phosphatase inhibitor cocktail) containing 200 μM tris(2-carboxylethyl)phosphine (TCEP), 15 mM MgCl_2,_ and benzonase (Millipore, for RIPA only) or 0.5% NP-40 buffer (50 mM Hepes, 150 mM NaCl, pH 7.4) containing protease inhibitors, phosphatase inhibitors, and 200 μM TCEP. Lysates in RIPA buffer were then sonicated, whereas lysates in NP-40 buffer were pipetted and centrifuged at 4600*g* for 1 min without sonication. Bradford assay was performed to measure the protein concentration, and the normalized cell lysates were denatured by adding lithium dodecyl sulfate supplemented with 50 mM DTT, followed by boiling at 90 °C for 5 min. Less than 30 μg of each lysate was loaded onto the 4 to 12% NuPAGE Bis-Tris gel (Thermo Fisher Scientific), followed by SDS-PAGE with MES running buffer. Following transfer to polyvinylidene fluoride membranes (0.45 μm or 0.2 μm, Millipore), blocking was performed with 5% nonfat milk at room temperature (RT) for 15 min and then incubated with primary antibodies diluted in Tris-buffered saline with tween20 (TBST) containing 2% BSA (1:1000) overnight at 4 °C. After the incubation with primary antibodies, membranes were washed with TBST and further incubated with fluorescent IRDye antibody (1:20,000) for 1 h at room temperature (RT). The membrane was then imaged using Chemidoc MP (Bio-Rad).

### Total proteomics analysis using TMTpro

The total proteomics analyses were performed based on the previously reported method (22). Briefly, HCT116 cells are plated into a 10 cm dish per condition, a total of 16 dishes. Twenty-four hours later, dTAGv1 (100 nM), HG (5 μM), or simvastatin (10 μM) were added to cells and incubated for 24 h. Cells were then washed with ice-cold PBS five times and lysed with 800 μl of RIPA buffer (50 mM Hepes, 150 mM NaCl, pH 7.6, 1% NP-40, 1% sodium deoxycholate, 0.1% SDS, 10 mM glycerophosphate, 10 mM sodium pyrophosphate, protease inhibitor cocktail, phosphatase inhibitor cocktail, pH 7.5). The lysate was then collected and sonicated three times, followed by Bradford assay to measure the protein concentration. The total protein concentration was adjusted to become 3 mg/ml throughout the samples using RIPA buffer. One hundred twenty-five micrograms of each protein extract was taken and reduced by incubation in the presence of 5 mM TCEP at 55 °C for 10 min. After cooling down to RT, the lysates were reacted with chloroacetamide solution (final conc. 20 mM) at RT for 15 min, followed by chloroform/methanol precipitation. Protein discs were resuspended in 100 mM EPPS (pH 8.5) containing 0.1% RapiGest and digested at 37 °C, 1200 rpm, overnight with Trypsin (100:1 protein-to-protease ratio). 16-plex tandem mass tag labeling of each sample was performed by adding 10 μl of the TMTpro reagent along with acetonitrile (ACN) to achieve a final ACN concentration of approximately 30% (v/v). Following incubation at RT for 1 h, the reaction was quenched with hydroxylamine to a final concentration of 1% (v/v) for 15 min. The TMTpro-labeled samples were pooled together at a 1:1 ratio. The sample was vacuum centrifuged to near dryness and subjected to C18 solid-phase extraction (50 mg, Sep-Pak, Waters). Dried TMTpro-labeled sample was resuspended in 100 μl of 10 mM NH_4_HCO_3_, pH 8.0, and off-line fractionated using basic pH reversed-phase HPLC (Agilent LC1260 Infinity II, equipped with a degasser and wavelength detector (set at 214 nm)). Peptides were subjected to a 60 min linear gradient from 8% to 40% ACN in 10 mM NH_4_HCO_3_, pH 8.0, at a flow rate of 0.6 ml/min over an Aeris peptide xb C18 column (Phenomenex; 2.6 μm, 100 Å, 4.6 mm ID and 250 mm in length). The 96 resulting fractions were then pooled in a noncontinuous manner into 24 fractions (as outlined in Fig. S5 of Paulo *et al.*’s paper) (63) for mass spectrometry analysis. Fractions were vacuum centrifuged to near dryness. Each consolidated fraction was desalted *via* Stage Tip, dried again *via* vacuum centrifugation, and reconstituted in 5% ACN, 1% formic acid for LC-MS/MS processing.

Mass spectrometry data were acquired using an Orbitrap Eclipse Tribrid mass spectrometer (Thermo Fisher Scientific) connected to an UltiMate 3000 RSLCnano UHPLC (Thermo Fisher Scientific). Peptides were separated on a 100 μm inner diameter microcapillary column packed in-house with ∼30 cm of HALO Peptide ES-C18 resin (2.7 μm, 160 Å, Advanced Materials Technology) with a gradient consisting of 5 % to 23% (0–75 min), 23 to 40% (75–110 min) (ACN, 0.1% FA) over a 120 min run at ∼500 nl/min. 3/10 of each fraction was loaded onto the column for analysis. Proteome analysis used Multi-Notch MS^3^-based TMT quantification (64), combined with Real Time Search analysis software (65, 66), and the FAIMS Pro Interface (using previously optimized 3 CV parameters) (67), to reduce ion interference. The scan sequence began with an MS^1^ spectrum (Orbitrap analysis; resolution 120,000 at 200 Th; mass range 400–1500 *m/z*; maximum injection time 50 ms; automatic gain control (AGC) target 4 × 10^5^). For MS^2^ analysis, precursors were selected based on a cycle time of 1.25 s/CV method (FAIMS CV = −40/−60/−80). MS^2^ analysis consisted of collision-induced dissociation (quadrupole ion trap analysis; rapid scan rate; AGC 1.0 × 10^4^; isolation window 0.5 Th; normalized collision energy 35; maximum injection time 35 ms). Monoisotopic peak assignment was used, and previously interrogated precursors were excluded using a dynamic window (180 s ± 10 ppm). Following the acquisition of each MS^2^ spectrum, a synchronous precursor selection API-MS^3^ scan was collected on the top 10 most intense ions b or y-ions matched by the online search algorithm in the associated MS^2^ spectrum (65, 66). MS^3^ precursors were fragmented by high-energy collision-induced dissociation and analyzed using the Orbitrap (normalized collision energy 45; AGC 2.5 × 10^5^; maximum injection time 200 ms, resolution was 50,000 at 200 Th). Closeout was set at two peptides per protein per fraction, so that MS^3^s were no longer collected for proteins having two peptide-spectrum matches (PSMs) that passed quality filters (66).

### Proteomics data analysis

Mass spectra were processed using a Comet-based (2020.01 rev. 4) software pipeline (68, 69). Spectra were first converted to mzXML and monoisotopic peaks were reassigned using Monocle software (70). MS^2^ spectra were matched with peptide sequences with a composite sequence database including the Human Reference Proteome UniProt database (Proteome ID: UP000005640, release 2022_01, Reviewed - Swiss-Prot canonical and isoforms entries only), as well as sequences of common contaminants. This database was concatenated with one composed of all protein sequences in the reverse order. Analysis was performed using a 50 ppm precursor ion tolerance. Static modifications included for Figure 2, *A* and *B*, TMTs (+229.163 Da), or for Figure 3, *B* and *D*, TMTpro tags (+304.207 Da) on lysine residues and peptide N termini, along with carbamidomethylation of cysteine residues (+57.021 Da). Oxidation of methionine residues (+15.995 Da) was set as a variable modification. PSMs were adjusted to a 1% false discovery rate (FDR) (71). PSM filtering was performed using a linear discriminant analysis (72), while considering the following parameters: Comet log expect, different sequence delta Comet log expect, missed cleavages, peptide length, charge state, precursor mass accuracy, and fraction of ions matched. For protein-level comparisons, PSMs were identified, quantified, and collapsed to a 1% peptide FDR and then collapsed further to a final protein-level FDR of 1% (73). To generate the smallest set of proteins required to account for all observed peptides, the principles of parsimony were applied. For TMT-based reporter ion quantitation, the summed signal-to-noise (S:N) ratio for each TMT channel was first extracted based on the closest matching centroid to the expected mass of the TMT reporter ion (integration tolerance of 0.003 Da for TMTpro, or 0.03 for TMT six plex). Isotopic impurities of the different TMT and TMTpro reagents provided by the manufacturer’s specifications were used to adjust reporter ion intensities. Proteins were quantified by summing reporter ion S:N measurements across all matching PSMs, resulting in a ‘‘summed S:N’’ measurement. For the total proteome, PSMs with poor quality, MS^3^ spectra missing 4 or more TMTpro reporter ion channels, isolation specificity below 0.5, or TMTpro reporter summed S:N ratio under 160, or with no MS^3^ spectra, were excluded from quantification. For the affinity-purification experiment, PSMs with poor quality, MS^3^ spectra missing 4 or more TMT reporter ion channels, isolation specificity below 0.3, or TMT reporter summed S:N ratio under 60, or with no MS^3^ spectra, were excluded from quantification.

Protein quantification values were exported for further analysis in Microsoft Excel and Perseus (74). In Perseus, a two-way Welch’s *t* test analysis was performed to compare two datasets, using the S0 parameter of 0.585 (a minimal fold change cutoff), and correction for multiple comparisons was achieved using the permutation-based FDR method, both of which are built-in functions of Perseus software. For immunoprecipitation mass-spectrometry (Fig. 2*B*), each TMT 6-plex channel was normalized to the streptavidin ion intensity, which was eluted after the hexafluoroisopropanol (HFIP) treatment. For whole-cell proteome analysis (Fig. 3*D*), each reporter ion channel was summed across all quantified proteins and normalized, assuming equal protein loading across all samples. The maximum and minimum TMT ratio quantifiable was capped at 100-fold. Organellar protein marker annotations were compiled using the proteins that had scored with confidence ‘‘very high’’ or ‘‘high’’ from a previously published HeLa dataset (75) and additional entries from manually curated literature.

GO analysis in Figure 3*E* was performed using the David GO (https://davidbioinformatics.nih.gov/summary.jsp). Briefly, 168 proteins mentioned in Figure 3*D* were analyzed, and the *Homo sapiens* whole genome default list was used as background. Among the functional analysis data, the cell compartment result was used for upregulated proteins. The maximum ease score (*p* value), a modified Fisher Exact *p* value as described on the David GO website, was used to rank the enrichment. GO terms with −Log10 *p* value >3.5 were plotted. Table S1 and Table S2 list all quantified proteins and the TMT reporter ratio associated with control channels used for quantitative analysis.

### MS/MS analysis of the HG-bound HMGCS1

HMGCS1 (9 μM, 125 μg total in 50 mM Hepes, 150 mM NaCl, 5% glycerol, pH 7.5, 25 μl reaction volume) was incubated with HG (100 μM final) or left untreated for 40 min at RT. Then, 175 μl of RIPA buffer containing 1% SDS and 3 mM TCEP was added and incubated for 5 min at RT. This was followed by incubation with iodoacetamide (20 mM final) for 15 min in the dark at RT. HMGCS1 was subsequently precipitated by adding 300 μl of cold water, 500 μl of methanol, and 100 μl of chloroform, vortexed, and centrifuged at 12,298*g*. The white protein pellet was washed once with cold methanol and dried. Since the tryptic peptide covering the catalytic cysteine of HMGCS1 (C129) is 46 amino acids long and could not be detected previously, we used Glu-C (Promega, 1.5 μg in 50 mM Tris–HCl buffer, 0.1% rapigest, 25 °C, overnight) for digestion, which is estimated to produce a 23-aa peptide. After confirming digestion through Coomassie staining of the SDS-PAGE gel, 5 μg of the sample was taken, desalted using a C18 stage tip, and an aliquot was analyzed by mass spectrometry. Analysis was carried out as for the total proteomics section, using an Orbitrap Eclipse Tribrid mass spectrometer. In brief, the sample was analyzed over a 75 min gradient (ACN, 0.1% formic acid), and the instrument was set up to survey MS^1^ scan using a single FAIMS CV (−40) (Orbitrap analysis, resolution 60,000 at 200 Th). MS^2^ analysis consisted of collision-induced dissociation (Orbitrap analysis; resolution 15,000 at 200 Th). Data were searched as described in the above data analysis section with the following modification. GluC instead of Trypsin was set as the digestion enzyme. Oxidation of methionine residues (+15.995 Da), carbamidomethylation of cysteine residues (+57.021 Da), and HG modification of cysteine residues (+324.192175 Da) were set as variable modifications. HG site localization was determined using the AScorePro algorithm (76, 77). AScore is a probability-based approach for posttranslational site localization. Specifically, a threshold of 13 corresponded to 95% confidence in site localization. Spectrum annotation for the HG modified catalytic site of HMGCS1 (Fig. S1*A*) was generated using IPSA (78).

### Affinity purification mass spectrometry analysis using hHG-biotin probe

HEK293T cells were seeded overnight before incubating with media containing 5 μM HG or vehicle control (DMSO) for 0.5 h. Then, 5 μM HG-biotin was introduced to the cells and incubated for an additional 2 h. Cells were washed with ice-cold PBS three times. The cells were then lysed with 200 μl of Hepes-NP-40 buffer (50 mM Hepes, 150 mM NaCl, pH 7.5, 0.25% NP-40). The lysate was centrifuged at 4600*g* for 1 min, and the supernatant was collected. The supernatant was then reduced by 5 mM TCEP (10 min, RT) and alkylated by chloroacetamide (20 mM final concentration, 15 min, RT). MeOH/CHCl_3_ precipitation was followed to remove any remaining HG-biotin in the cell lysates. The white protein disk was then resuspended in 2% SDS (50 mM Hepes 150 mM NaCl, pH7.2, 2.5 mM TCEP) and sonicated once with a tip sonicator and then diluted in Hepes-NP-40 buffer (final SDS concentration is <0.5%). Subsequently, 10 μl of high-capacity streptavidin was added to the cell lysates and incubated for 2 h at RT. Flow-through was stored for the quality control immunoblots, and the beads were washed with 2X RIPA, 3X 2% SDS, 2X RIPA, and 3X PBS. The beads were transferred onto polytetrafluoroethylene membrane filter cups and washed twice with water. Completely dried beads were resuspended in 50 μl of HFIP, incubated for 5 min with shaking, and eluted by spinning at 2000*g*. Fifty microliters of HFIP was added to the beads again, and the eluates were combined and dried in speed vac. Ten percent of this was analyzed by immunoblotting, whereas the remaining 90% was proceeded for 6-plex TMT labeling following the protocol described in the proteomics sample preparation section. The sample was then fractionated according to the manufacturer’s instructions using high pH reversed-phase peptide fractionation kit (Pierce Cat# 84868) for a final 6 fractions and subjected to C18 StageTip desalting prior to MS analysis.

### Protein expression and purification

For bacterial expression of proteins, BL21 cells were transformed with the pET60b-HMGCS1-TEV-6His vector, and cultured in 1 L of Terrific broth at 37 °C to an absorbance (A_600_) ∼1.0, and induced with 0.5 mM IPTG overnight at 18 °C. Harvested cells were homogenized in lysis buffer (50 mM Hepes, pH 7.5, 150 mM NaCl, 1% Triton-X, 0.06% beta-mercaptoethanol, 1 tablet of protease inhibitor) and centrifuged at 15,000*g*. The supernatant was applied to the nickel-nitrilotriacetic acid resin (1 ml) and washed with high salt buffer (50 mM Hepes, 500 mM NaCl, 1 M urea, 20 mM imidazole, pH 7.5) and low-salt buffer (50 mM Hepes, 150 mM NaCl, pH 7.5). Then, the resin was eluted with elution buffer (50 mM Hepes, 150 mM NaCl, 500 mM imidazole, pH 7.5). The eluent was applied to a Superdex 75 column, pre-equilibrated with a low-salt buffer. The final protein yield was 5 mg/l of culture.

### In-gel fluorescent analysis with HG-fluorescence probes

Recombinant HMGCS1 protein (1 μg) or cell lysates were incubated with 5 μM HG-FITC (HG-FL) or HG-TMR probe at 37 °C for 1 h. The labeling reaction was stopped by adding 4X lithium dodecyl sulfate containing DTT and boiling at 90 °C for 5 m. The samples were subjected to SDS-PAGE, and the fluorescent signal in the gel was detected by Chemidoc MP (Bio-Rad).

### Cell viability assay

Cells viability was evaluated using WST1-based colorimetric assay (Roche). A total of 2 × 10^4^ cells in 100 μl of culture medium were seeded for each well of 96-well plates. Next day, the cells were treated with culture media containing various amounts of simvastatin and 50 nM of dTAGv1. After 24 h, cells were incubated with WST-1 solution, which was diluted to 1:10 with culture medium in the cell culture incubator. After 4 h, the absorbance of samples was measured using a microplate reader. The wavelength to measure the absorbance of formazan product from viable cells was 450 nm, and the reference wavelength was 690 nm.

### Colony formation assay

To evaluate the ability of single cancer cells to form a colony, cells were plated at a concentration of 1500 cells/well onto 6-well plates (day 0). At day 5 and day 7, the cells were treated with the corresponding chemicals. On day 12, the cells were stained with a crystal violet working solution, which contains 0.5% crystal violet and 4% paraformaldehyde in PBS. Numbers of colonies were counted for each well (n = 2 or 3) and presented as mean ± SD.

### Animal experiments

Animal experiments were performed in compliance with protocols approved by the Research Animal Resource Center and Institutional Animal Care and Use Committee at Memorial Sloan Kettering Cancer Center. Twenty athymic nude mice (nu/nu athymic mice, Charles River Laboratory) were implanted with HCT116 HMGCS1-FKBP12^F36V^ homozygous KI cells through subcutaneous injection of 2 × 10^6^ cells per mouse. The treatment was initiated as the tumor size became 200 to 250 mm^3^. Four groups of mice were administered with two treatment regimens over a 2-week interval, including saline control, 5 mg/kg simvastatin three times per week *via* oral gavage, 5 mg/kg dTAGv1 weekly *via* intraperitoneally injected, and the combination of both simvastatin and dTAGv1. PBS is used as a vehicle for both simvastatin and dTAGv1. Tumor growth was monitored and recorded twice a week for 40 days, and five mice were used per condition.

### Quantification and statistical analysis

*p* values in the volcano plots of the proteomics data were analyzed by two-sided Welch’s *t* test, which was adjusted for multiple comparisons. Comparisons of the quantified data (immunoblotting, colony formation, tumor spheroids) were performed by unpaired Student’s *t* test; Statistical significance was judged based on *p* values; ∗*p* < 0.05; ∗∗*p* < 0.01; and ∗∗∗*p* < 0.001.

## Data availability

• All unique identifiers and web links for publicly available datasets are presented within the main text or the supplementary materials. The proteomic data generated in this paper has been deposited in the MassIVE repository with the dataset identifier: MSV000098111.
• This paper does not report any original code.
• Any additional information required to reanalyze the data reported in this paper is available from the lead contact upon request.

## Supporting information

This article contains supporting information.

## Conflict of interest

The authors declare that they have no conflicts of interest with the contents of this article.
