# Supplementary material for: Activity-based probes and chemical proteomics uncover the biological impact of targeting HMG-CoA Synthase 1 in the mevalonate pathway
Source: J Biol Chem. 2025 Sep 3;301(10):110660. doi: 10.1016/j.jbc.2025.110660 (PMC12514577; doi:10.1016/j.jbc.2025.110660)
Supplement: Supporting Information [file mmc1.pdf]

Synthetic Procedures

General Details

All reagents were purchased from commercial sources and used as received unless stated otherwise. Analytical TLC has been performed on Silicycle SiliaPlate TLC plates and visualized under UV light (254 nm) or by staining with potassium permanganate. Flash chromatography was carried out on Sigma silica gel (200-300 mesh). All new compounds were characterized by UPLC-SQD-LC-MS (waters). (4.6 mm ×150 mm 5 μm C18 column; 10 μL injection; 10-100% CH_3CN/H_2O, linear-gradient, with constant 0.1% v/v TFA additive; 8 min run; ESI; positive ion mode; UV detection at 190-500 nm)

Synthesis of Hymeglusin-Fluorescein (HG-FL).

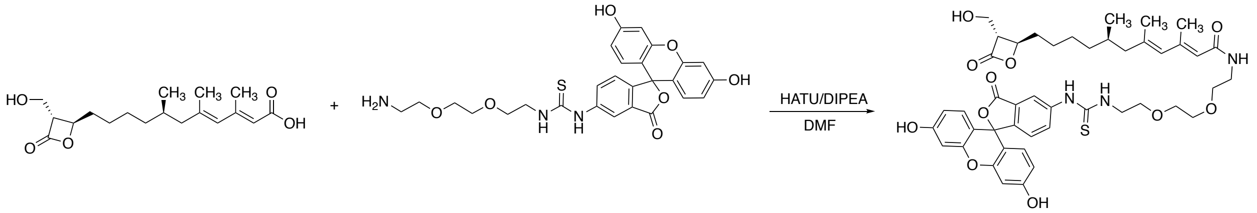

In a 5-ml round bottom flask, hymeglusin (0.5 mg, 1.5 μM) was dissolved in 1 ml DMF. *N, N*-diisopropylethylamine (DIPEA, 2 μL, 10 μM), and Hexafluorophosphate Azabenzotriazole Tetramethyl Uronium (HATU, 1 mg, 2.6 μM) were added to the solution. After stirring for 30 min at room temperature, fluorescein-PEG2-amine (1 mg, 1.5 μM) was added, and the solution was stirred overnight. The reaction mixture was poured into the 5 ml ether to precipitate the crude product. The crude product was purified via a microscale silica column eluting with 10-20% Methanol in DCM and yielded as an orange solid. HG-FL (1.0 mg, 77%) **MS (ESI)** m/z calcd for $C_{45}H_{52}N_3O_{11}S$ (842.3339); found 842.3358.

Synthesis of Hymeglusin-Rhodamine (HG-TMR).

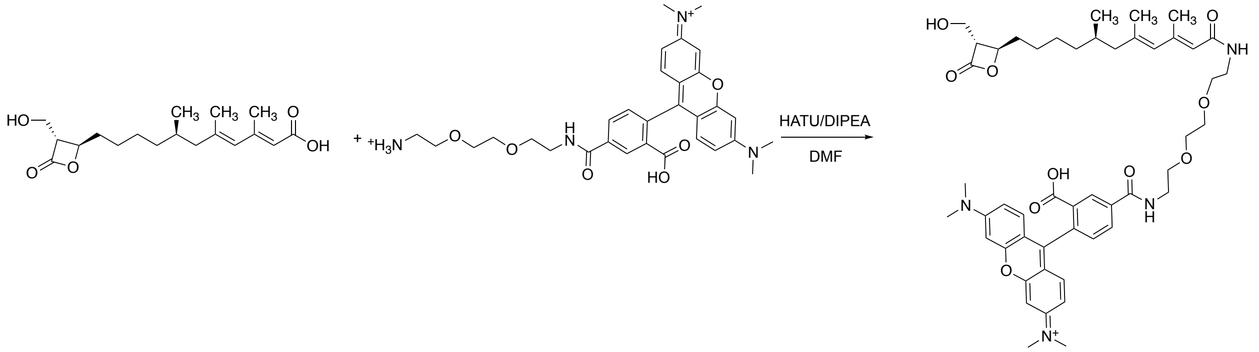

In a 5-ml round bottom flask, hymeglusin (0.5 mg, 1.5 μM) was dissolved in 1 ml DMF, and then DIPEA (2 μL, 10 μM) and HATU (1 mg, 2.6 μM) were added to the solution. After stirring for 30 min at room temperature, TMR-PEG2-amine (1 mg, 1.5 μM) was added, and the solution was stirred overnight. The reaction mixture was poured into the 5 ml ether to precipitate the crude product. The crude product was purified via a microscale silica column eluting with 10-20% Methanol in DCM and yielded as an orange solid. HG-TMR (1.0 mg, 70%) **MS (ESI)** m/z calcd for $C_{49}H_{63}N_4O_{10}$ (867.4539); found 867.4503.

Synthesis of Hymeglusin-Biotin (HG-Biotin).

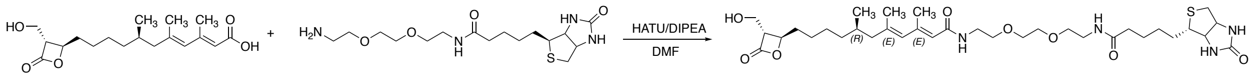

In a 5-ml round bottom flask, hymeglusin (0.5 mg, 1.5 μM) was dissolved in 1 ml DMF, and then DIPEA (2 μL, 10 μM) and HATU (1 mg, 2.6 μM) were added to the solution. After stirring for 30 min at room temperature, Biotin-PEG2-amine (0.6 mg, 1.5 μM) was added, and the solution was stirred overnight. The reaction mixture was poured into the 5 ml ether to precipitate the crude product. The crude product was purified via a microscale silica column eluting with 20-30% Methanol in DCM and yielded as a white solid. HG-Biotin (0.5 mg, 48%) **MS (ESI)** m/z calcd for $C_{34}H_{56}N_4O_8SNa$ (703.3717); found 703.3718.

Synthesis of Hymeglusin-PROTAC (HG-PROTAC) 1-5.

CRBN recruiting ligand.

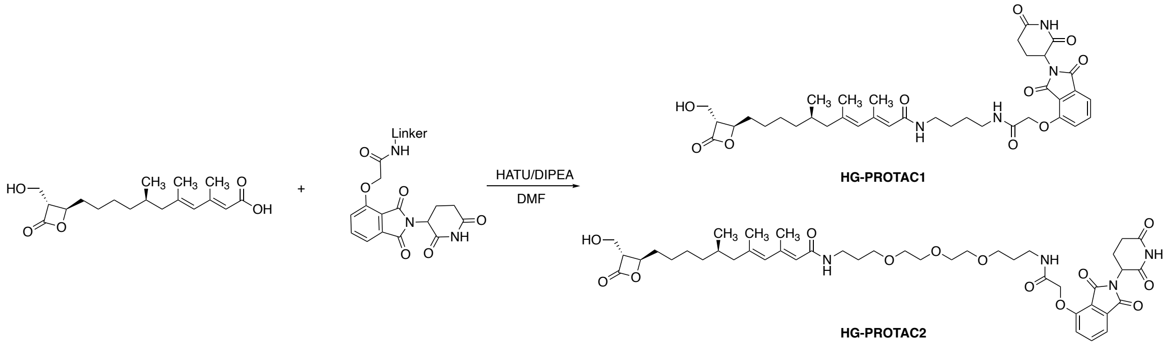

In a 5-ml round bottom flask, hymeglusin (0.5 mg, 1.5 μM) was dissolved in 1 ml DMF, and then DIPEA (2 μL, 10 μM) and HATU were added to the solution. After stirring for 30 minutes at room temperature, Phenthalimide-linker amines (1 mg, 1.5 μM) were added, stirring the solution overnight. The reaction mixture was poured into the 5 ml ether to precipitate the crude product. The crude product was purified via a microscale silica column eluting with 5-10% Methanol in DCM and yielded as a white solid. HG-PROTAC1 (0.5 mg, 46%) **MS (ESI)** m/z calcd for $C_{37}H_{48}N_4O_{10}Na$ (731.3268); found 731.3236. HG-PROTAC2 (0.5 mg, 38%) **MS (ESI)** m/z calcd for $C_{43}H_{60}N_4O_{13}Na$ (863.4055); found 863.4046.

VHL recruiting ligand.

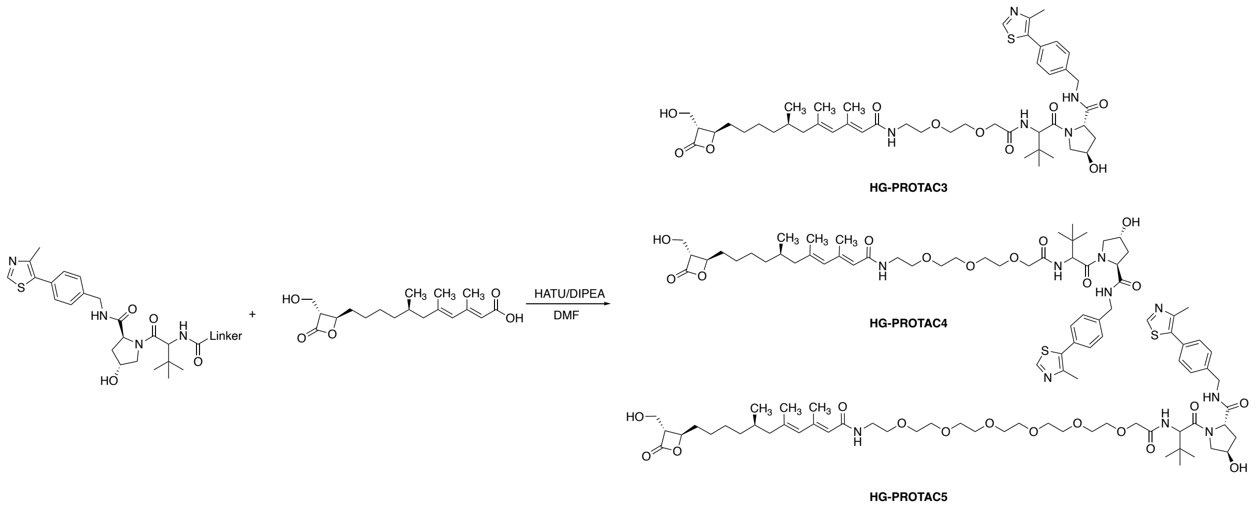

Hymeglusin (0.5 mg, 1.5 μM) was dissolved in 1 ml DMF, and then DIPEA (2 μL, 10 μM) and HATU (1 mg, 2.6 μM) were added to the solution. After stirring for 30 min at room temperature, VHL-linker amine (1 mg, 1.5 μM) was added, and the solution was stirred overnight. The reaction mixture was poured into the 5 ml ether to precipitate the crude product. The crude product was purified via a microscale silica column eluting with 5-10% Methanol in DCM and yielded as a white solid. HG-PROTAC3 (1.0 mg, 72%) **MS (ESI)** m/z calcd for $C_{46}H_{67}N_{5}O_{10}SNa$ (904.4506); found 904.4470. HG-PROTAC4 (1.1 mg, 77%) **MS (ESI)** m/z calcd for $C_{48}H_{71}N_{5}O_{11}SNa$ (948.4768); found 948.4752. HG-PROTAC5 (1.0 mg, 62%) **MS (ESI)** m/z calcd for $C_{54}H_{83}N_{5}O_{14}SNa$ (1080.5555); found 1080.5542.

UV trace and High-resolution Mass-spectrometry spectra

Hymeglusin-Fluorescein

UV trace (190-500 nm)

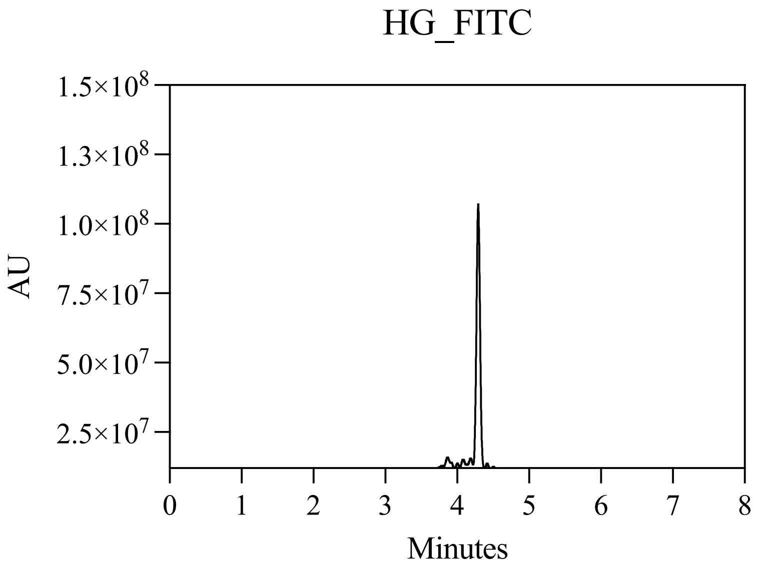

HiRes-MS:

Single Mass Analysis
Tolerance = 5.0 PPM / DBE: min = -1.5, max = 50.0
Element prediction: Off
Number of isotope peaks used for i-FIT = 3

Monoisotopic Mass, Even Electron Ions
84 formula(e) evaluated with 1 results within limits (up to 50 closest results for each mass)
Elements Used:
C: 0-45 H: 0-55 N: 0-3 O: 0-11 S: 0-1

An Heeseon
Liang S
C45H54N3O11S
HG_FTLC 59 (1.330) Cm (59:61)

NMR Analytical Core Facility
LCT Premier XE

20-Mar-2025
1::9::7

1: TOF MS ES-
3.82e+003

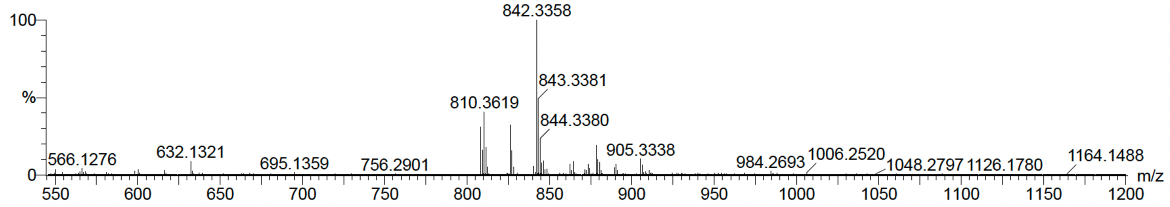

Mass	Calc. Mass	mDa	PPM	DBE	i-FIT	i-FIT (Norm)	Formula
Minimum:				-1.5			
Maximum:		5.0	5.0	50.0			
842.3358	842.3323	3.5	4.2	21.5	130.9	0.0	C45 H52 N3 O11 S

Hymeglusin-TMR

UV trace (190-500 nm)

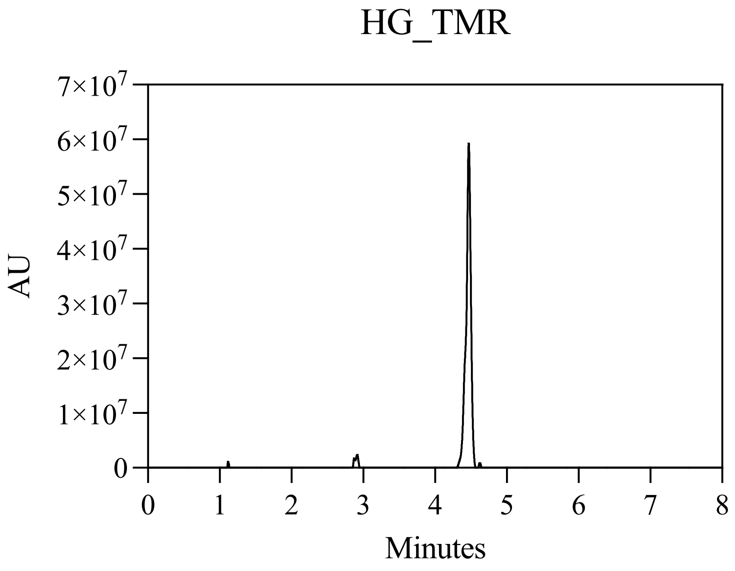

HiRes-MS:

Single Mass Analysis
Tolerance = 5.0 PPM / DBE: min = -1.5, max = 50.0
Element prediction: Off
Number of isotope peaks used for i-FIT = 3

Monoisotopic Mass, Even Electron Ions
46 formula(e) evaluated with 1 results within limits (up to 50 closest results for each mass)
Elements Used:
C: 0-49 H: 0-64 N: 0-4 O: 0-10

An Heeseon
Liang S
$C_{49}H_{63}N_4O_{10}$
HG_TMR 31 (0.697) Cm (29:32)

NMR Analytical Core Facility
LCT Premier XE

21-Mar-2025
12:36:24

1: TOF MS ES+
7.90e+002

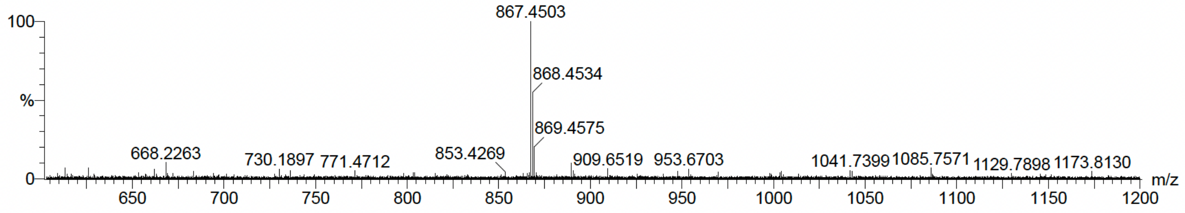

Minimum:				-1.5			
Maximum:		5.0	5.0	50.0			
Mass	Calc. Mass	mDa	PPM	DBE	i-FIT	i-FIT (Norm)	Formula
867.4503	867.4544	-4.1	-4.7	20.5	108.1	0.0	C49 H63 N4 O10

Hymeglusin-Biotin

UV trace (190-500 nm)

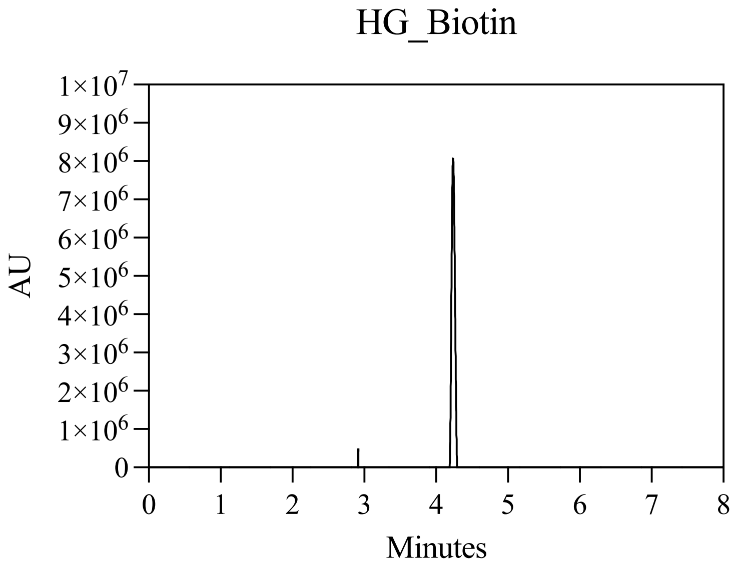

HiRes-MS:

Single Mass Analysis
Tolerance = 5.0 PPM / DBE: min = -1.5, max = 50.0
Element prediction: Off
Number of isotope peaks used for i-FIT = 3

Monoisotopic Mass, Even Electron Ions
210 formula(e) evaluated with 1 results within limits (up to 50 closest results for each mass)
Elements Used:
C: 0-34 H: 0-58 N: 0-5 O: 0-8 Na: 0-1 S: 0-1

An Heeseon
Liang S
C34H56N4O8S
CS_2_86_HG_Bio 41 (0.934) Cm (41:44)

NMR Analytical Core Facility
LCT Premier XE

20-Mar-2025
1::3::5

1: TOF MS ES+
2.14e+004

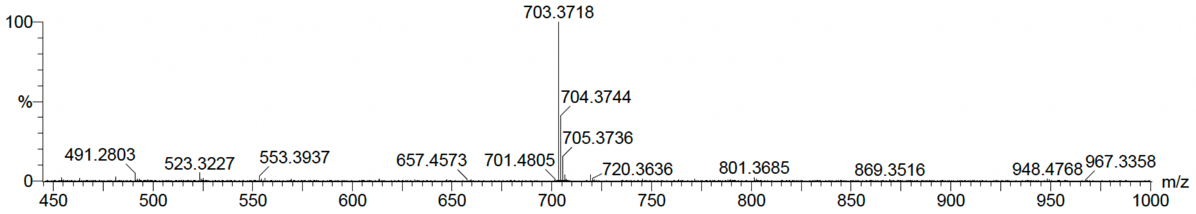

Minimum:				-1.5			
Maximum:		5.0	5.0	50.0			
Mass	Calc. Mass	mDa	PPM	DBE	i-FIT	i-FIT (Norm)	Formula
703.3718	703.3717	0.1	0.1	8.5	218.5	0.0	C34 H56 N4 O8 Na S

HG-PROTAC1

UV trace (190-500 nm)

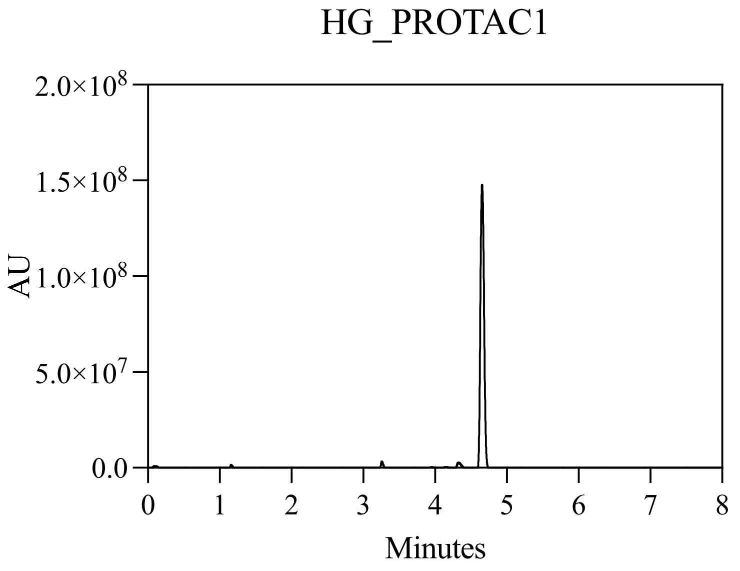

HiRes-MS:

Single Mass Analysis
Tolerance = 5.0 PPM / DBE: min = -1.5, max = 50.0
Element prediction: Off
Number of isotope peaks used for i-FIT = 3

Monoisotopic Mass, Even Electron Ions
133 formula(e) evaluated with 1 results within limits (up to 50 closest results for each mass)
Elements Used:
C: 0-37 H: 0-50 N: 0-4 O: 0-13 Na: 0-1

An Heeseon
Liang S
C37H48N4O10
HG_PROTAC_1 49 (1.117) Cm (49:53)

NMR Analytical Core Facility
LCT Premier XE

20-Mar-2025
1::3::2

1: TOF MS ES+
8.12e+003

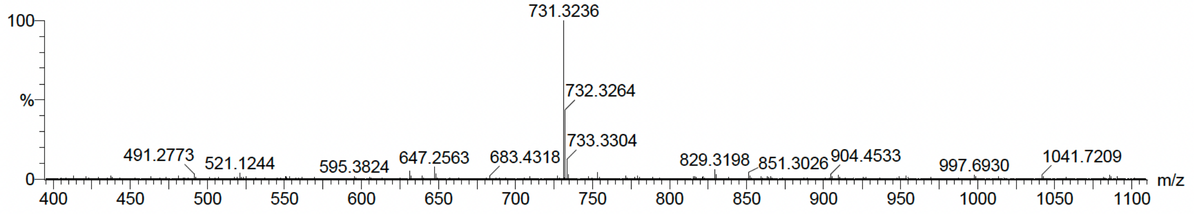

Minimum:				-1.5			
Maximum:		5.0	5.0	50.0			
Mass	Calc. Mass	mDa	PPM	DBE	i-FIT	i-FIT (Norm)	Formula
731.3236	731.3268	-3.2	-4.4	15.5	201.6	0.0	C37 H48 N4 O10 Na

HG-PROTAC2

UV trace (190-500 nm)

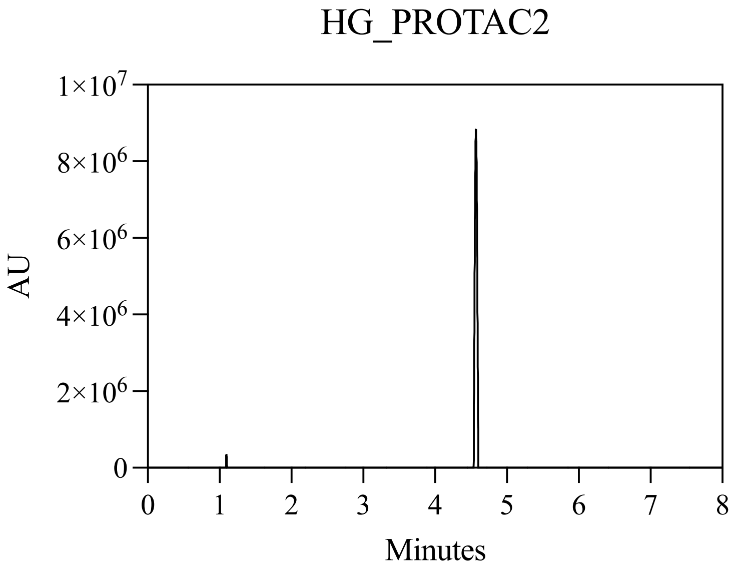

HiRes-MS:

Single Mass Analysis
Tolerance = 5.0 PPM / DBE: min = -1.5, max = 50.0
Element prediction: Off
Number of isotope peaks used for i-FIT = 3

Monoisotopic Mass, Even Electron Ions
132 formula(e) evaluated with 1 results within limits (up to 50 closest results for each mass)
Elements Used:
C: 0-43 H: 0-61 N: 0-4 O: 0-13 Na: 0-1

An Heeseon
Liang S
C43H60N4O13
HG_PROTAC_2 61 (1.389) Cm (61:64)

NMR Analytical Core Facility
LCT Premier XE

20-Mar-2025
1::4::0

1: TOF MS ES+
6.86e+003

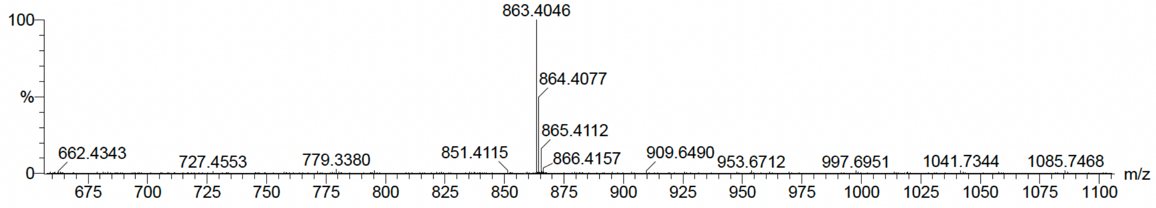

	Mass	Calc. Mass	mDa	PPM	DBE	i-FIT	i-FIT (Norm)	Formula
Minimum:					-1.5			
Maximum:			5.0	5.0	50.0			
	863.4046	863.4055	-0.9	-1.0	15.5	131.2	0.0	C43 H60 N4 O13 Na

HG-PROTAC3

UV trace (190-500 nm)

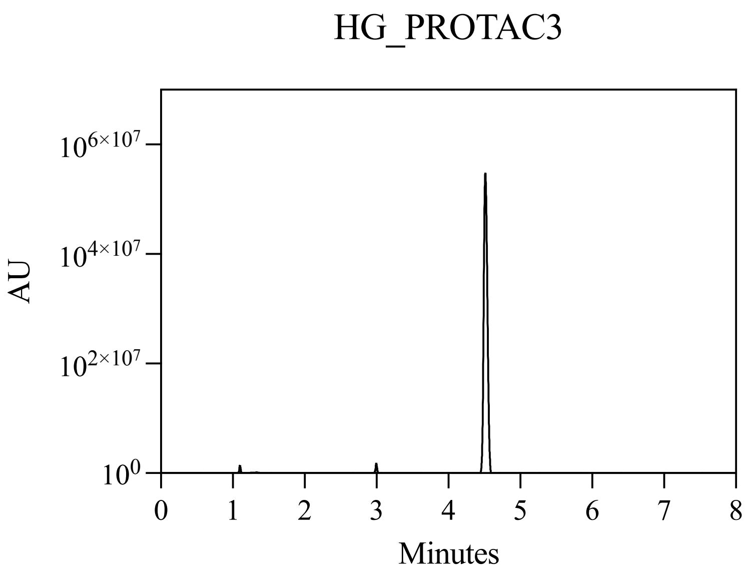

HiRes-MS:

Single Mass Analysis
Tolerance = 5.0 PPM / DBE: min = -1.5, max = 50.0
Element prediction: Off
Number of isotope peaks used for i-FIT = 3

Monoisotopic Mass, Even Electron Ions
315 formula(e) evaluated with 1 results within limits (up to 50 closest results for each mass)
Elements Used:
C: 0-46 H: 0-68 N: 0-5 O: 0-13 Na: 0-1 S: 0-1

An Heeseon
Liang S
C46H67N5O10S
HG_PROTAC_3 58 (1.315) Cm (58:61)

NMR Analytical Core Facility
LCT Premier XE

20-Mar-2025
1::9::8

1: TOF MS ES+
2.79e+003

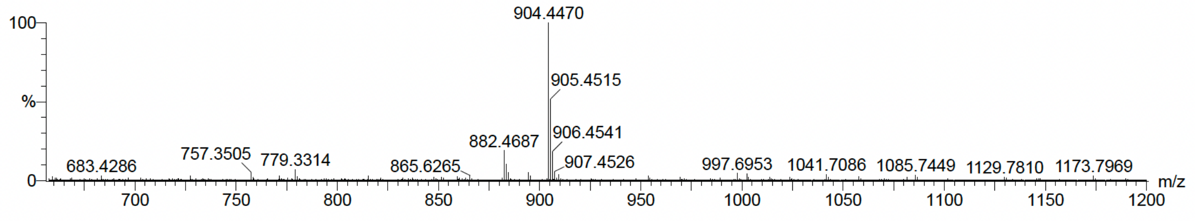

Minimum:				-1.5			
Maximum:		5.0	5.0	50.0			
Mass	Calc. Mass	mDa	PPM	DBE	i-FIT	i-FIT (Norm)	Formula
904.4470	904.4506	-3.6	-4.0	15.5	104.3	0.0	C46 H67 N5 O10 Na S

HG-PROTAC4

UV trace (190-500 nm)

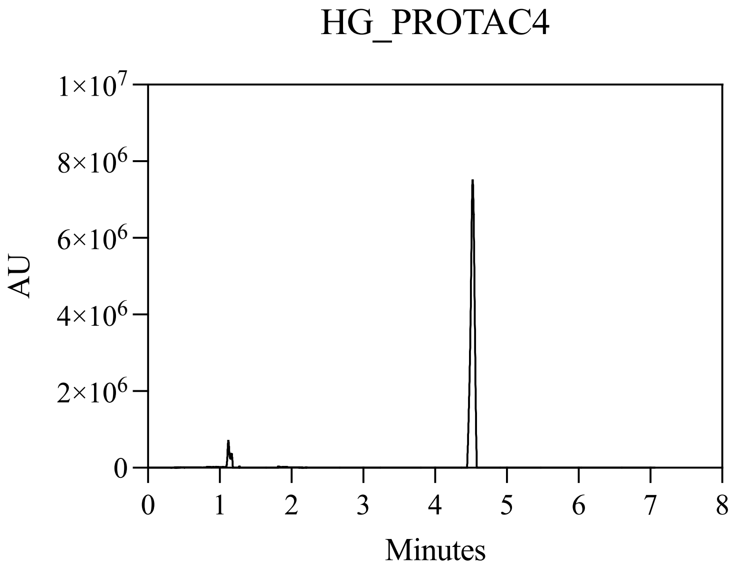

HiRes-MS:

Single Mass Analysis
Tolerance = 5.0 PPM / DBE: min = -1.5, max = 50.0
Element prediction: Off
Number of isotope peaks used for i-FIT = 3

Monoisotopic Mass, Even Electron Ions
279 formula(e) evaluated with 1 results within limits (up to 50 closest results for each mass)
Elements Used:
C: 0-48 H: 0-72 N: 0-5 O: 0-11 Na: 0-1 S: 0-1

An Heeseon
Liang S
C48H71N5O11S
HG_PROTAC_4 64 (1.441) Cm (61:64)

NMR Analytical Core Facility
LCT Premier XE

20-Mar-2025
1::7::1

1: TOF MS ES+
1.06e+004

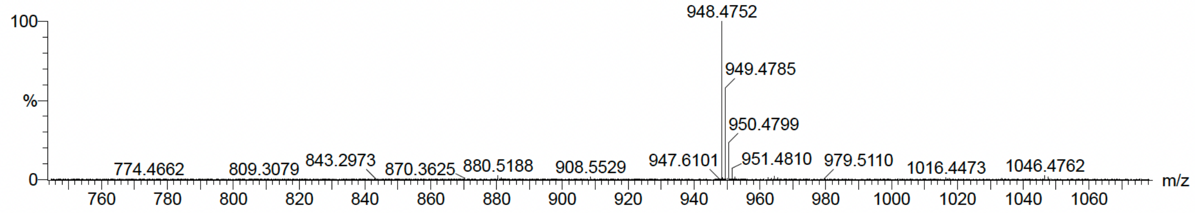

Minimum:				-1.5			
Maximum:		5.0	5.0	50.0			
Mass	Calc. Mass	mDa	PPM	DBE	i-FIT	i-FIT (Norm)	Formula
948.4752	948.4769	-1.7	-1.8	15.5	132.1	0.0	C48 H71 N5 O11 Na S

HG-PROTAC5

UV trace (190-500 nm)

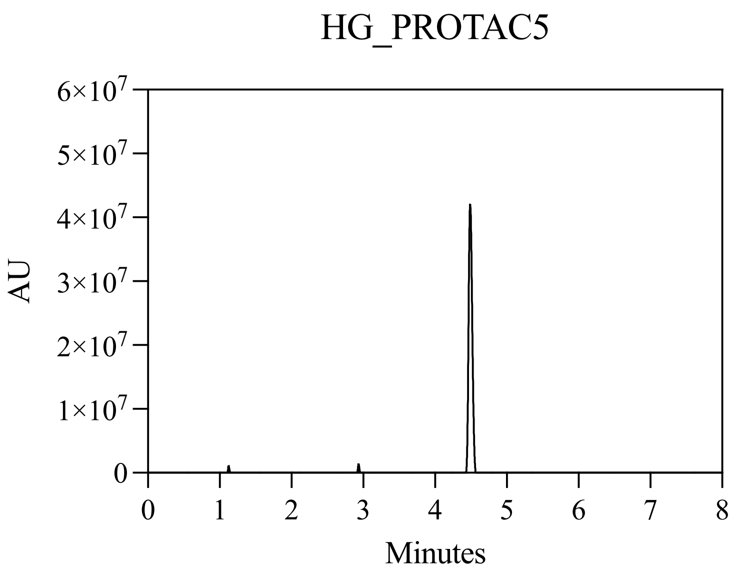

HiRes-MS:

Single Mass Analysis
Tolerance = 5.0 PPM / DBE: min = -1.5, max = 50.0
Element prediction: Off
Number of isotope peaks used for i-FIT = 3

Monoisotopic Mass, Even Electron Ions
351 formula(e) evaluated with 1 results within limits (up to 50 closest results for each mass)
Elements Used:
C: 0-54 H: 0-84 N: 0-5 O: 0-14 Na: 0-1 S: 0-1

An Heeseon
Liang S
C54H83N5O14S
HG_PROTAC_5 38 (0.861) Cm (38:41)

NMR Analytical Core Facility
LCT Premier XE

20-Mar-2025
1::3::6

1: TOF MS ES+
4.32e+004

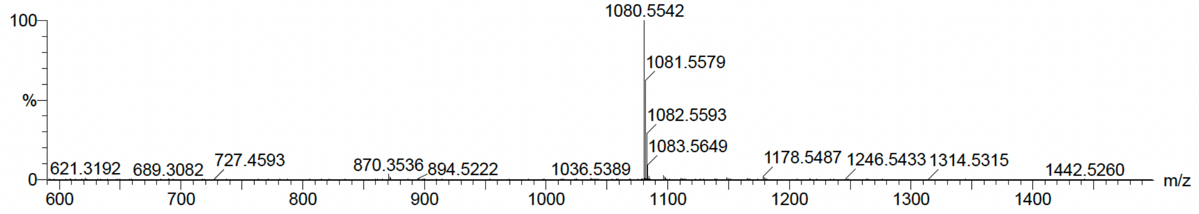

Minimum:				-1.5			
Maximum:		5.0	5.0	50.0			
Mass	Calc. Mass	mDa	PPM	DBE	i-FIT	i-FIT (Norm)	Formula
1080.5542	1080.5555	-1.3	-1.2	15.5	202.4	0.0	C54 H83 N5 O14 Na S
