# Supplementary material for: Activity-based probes and chemical proteomics uncover the biological impact of targeting HMG-CoA Synthase 1 in the mevalonate pathway
Source: J Biol Chem. 2025 Sep 3;301(10):110660. doi: 10.1016/j.jbc.2025.110660 (PMC12514577; doi:10.1016/j.jbc.2025.110660)
Supplement: Supporting Figures [file mmc2.pdf]

Figure S1

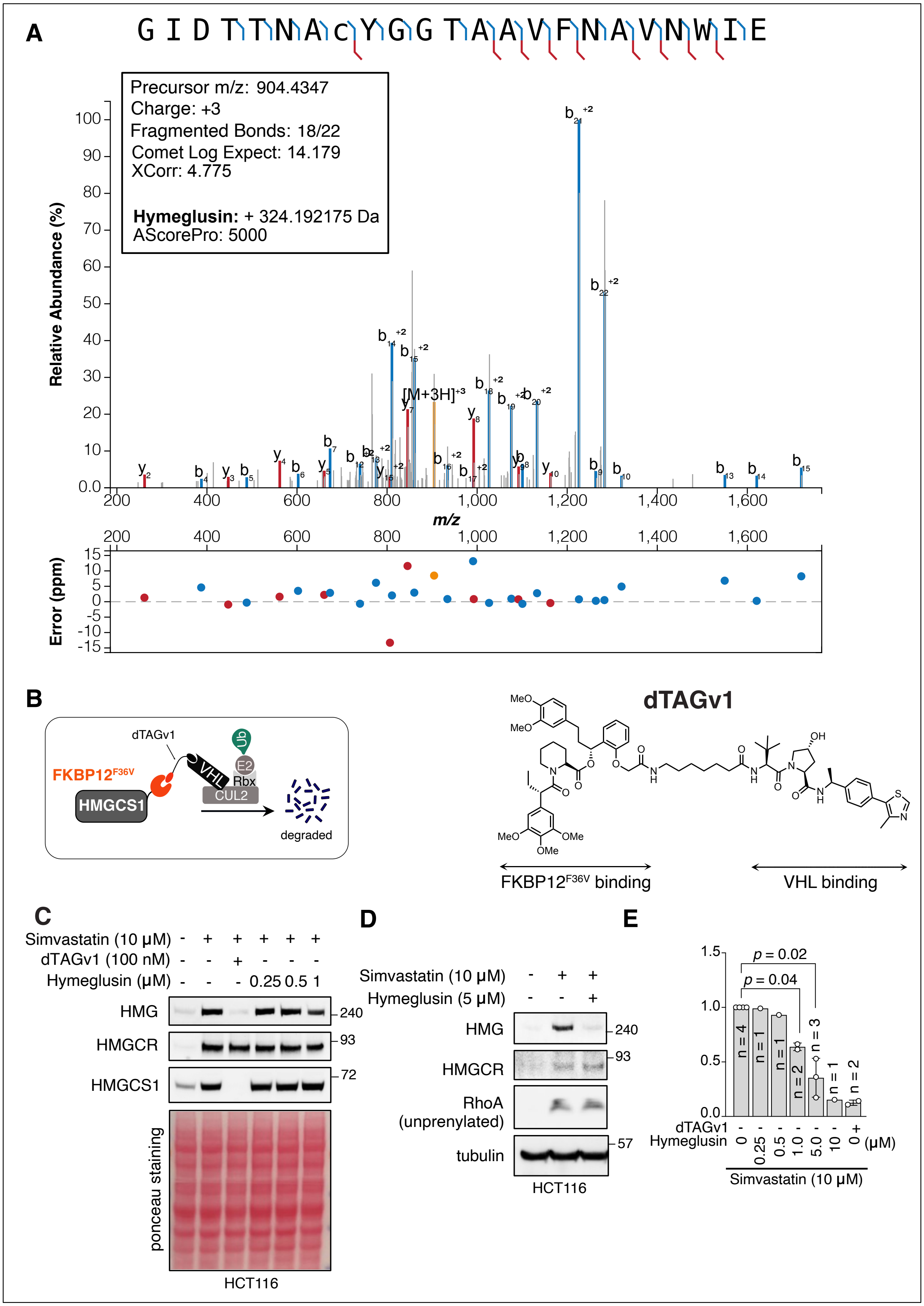

A
G I D T T N A c Y G G T A A V F N A V N W I E
Precursor m/z: 904.4347
Charge: +3
Fragmented Bonds: 18/22
Comet Log Expect: 14.179
XCorr: 4.775
Hymeglusin: + 324.192175 Da
AScorePro: 5000
Relative Abundance (%)
m/z
Error (ppm)
[M+3H]+3
B
dTAGv1
FKBP12F36V
HMGCS1
VHL
Ub
E2
Rbx
CUL2
degraded
FKBP12F36V binding
VHL binding
C
Simvastatin (10 µM)
dTAGv1 (100 nM)
Hymeglusin (µM)
HMG
HMGCR
HMGCS1
ponceau staining
HCT116
D
Simvastatin (10 µM)
Hymeglusin (5 µM)
HMG
HMGCR
RhoA (unprenylated)
tubulin
HCT116
E
p = 0.02
p = 0.04
dTAGv1
Hymeglusin
(µM)
Simvastatin (10 µM)

SI FIGURE 1. (Related to Figure 1).

(A) MS/MS spectrum annotation of human HMGCS1 peptide (residues 122-144) modified with Hymeglusin at C129 position. The peptide sequence (top) shows the locations of matched fragment ions, the middle displays the annotated mass spectrum, and the bottom visualizes the mass error in parts-per-million for all matched fragment ions.

(B) The schematic of the dTAG system (left) and chemical structure of the dTAGv1 ligand (right). This bifunctional ligand can bring VHL closer to the FKBP12^{F36V} mutant.

(C,D) Assessing the changes in HMG-positive band intensity after treatment with different concentrations of Hymeglusin in HCT116 cells.

(E) Quantification of HMG-positive band intensity under different Hymeglusin or dTAGv1 treatment conditions. n = 1, 2, 3, or 4 biological replicates. Mean ± s.d.

Figure S2

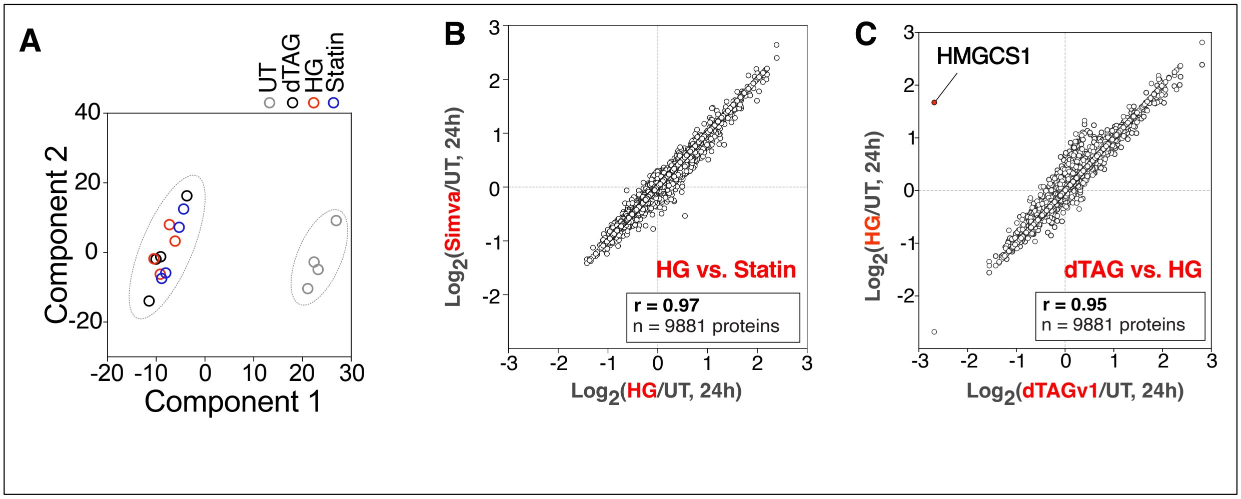

SI FIGURE 2. Global proteomic changes induced upon MVP inhibition (Related to Figure 3).

(A) Principal component analysis (PCA) of the total proteomics dataset, prepared as shown in Fig. 3B, indicates that dTAG, HG, and Statin-treated cells are grouped together.

(B,C) 9881 proteins were plotted for the log_2-ratio of (Simvastatin/DMSO) against the log_2-ratio of (HG/DMSO) for panel B, and log_2-ratio of (Hymeglusin/DMSO) against the log_2-ratio of (dTAGv1/DMSO) for panel C. N = 4 replicates.

Figure S3

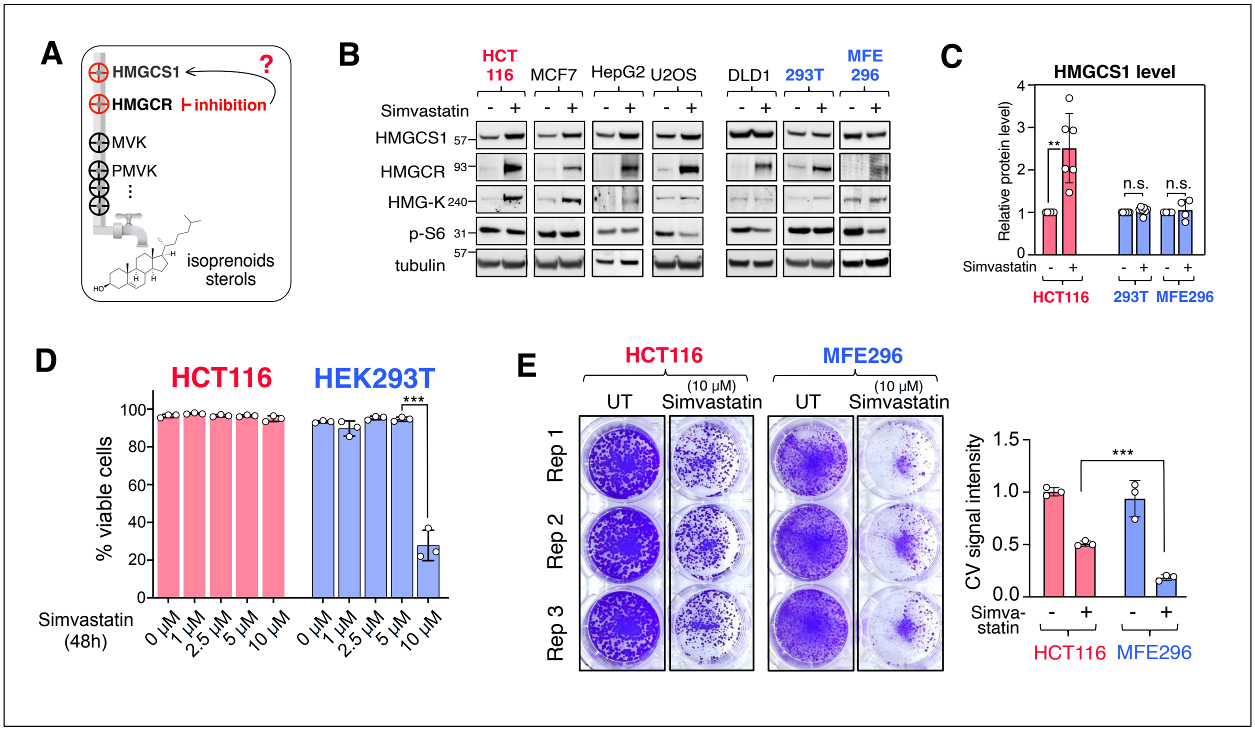

SI FIGURE 3. Relation between HMGCS1 upregulation and statin resistance (Related to Figure 4).

(A) A schematic showing the relationship between HMGCR inhibition and HMGCS1 up-regulation.

(B) The level of HMGCS1 upon Simvastatin treatment for 24 hours was assessed in seven different cell lines using immunoblotting analysis. Indicated cells were treated with Simvastatin (10 μM, 24h), followed by immunoblotting with indicated antibodies.

(C) The relative level of HMGCS1 protein was quantified from the experimental replicates shown in panel B, with n > 4 replicates. Mean ± s.d.

(D) Flow cytometry analysis of DAPI-stained versus unstained cells was conducted to evaluate cell viability after treating indicated cells with increasing concentrations of Simvastatin for 48 hours. Mean ± s.d. n = 3 replicates.

(E) Colony formation assay with or without Simvastatin treatment was performed on MFE296 and HCT116 cell lines. The total signal intensity per plate, relative to the untreated condition, is presented on the right. Mean ± s.d. n = 3 replicates.

Figure S4

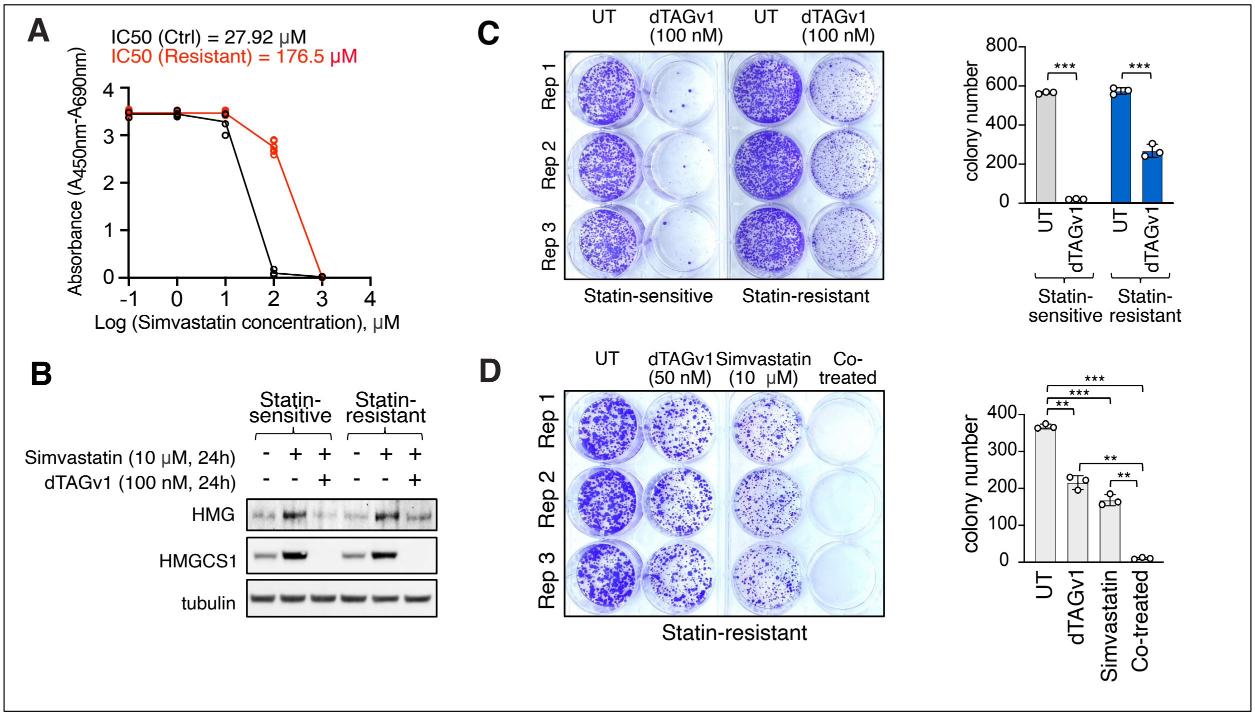

SI FIGURE 4. Combined targeting of HMGCS1 and HMGCR shows synergistic anti-proliferative effects on statin-resistant HCT116 cells (Related to Figure 4).

(A) Cell survival of wild-type and statin-resistant HCT116 upon statin treatment was measured using the cell proliferation reagent WST-1. Statins-resistant HCT116 cells were acquired by treating the cells with increasing concentrations of Simvastatin (5 to 30 μM) over a 4-month period.

(B) Immunoblotting analysis of WT and statin-resistant HCT116 shows the expected response to HMGCS1 degradation or HMGCR inhibition.

(C) Colony formation assay shows that the statin-resistant cells exhibit reduced sensitivity to HMGCS1 degradation. The corresponding cells were treated with dTAGv1 (100 nM) for 5 days prior to staining with crystal violet. Colony numbers were presented on the right. Mean ± s.d. n = 3 replicates.

(D) Co-treatment with Simvastatin (10 μM) and dTAGv1 (50 nM) shows enhanced cytotoxic effects on statin-resistant HCT116 cells. Colony numbers were presented on the right. Mean ± s.d. n = 3 replicates.

Figure S5

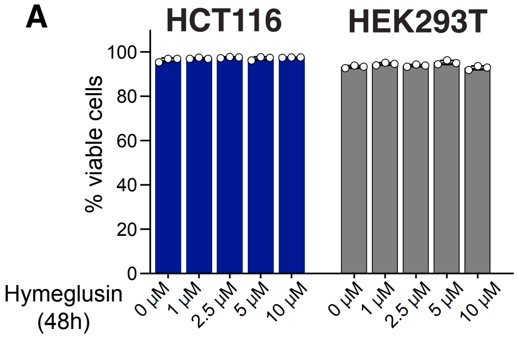

SI FIGURE 5. Limited efficacy of Hymeglusin on cell proliferation (Related to Figure 5).

(A) HEK293T and HCT116 cells were treated with increasing concentrations of Hymeglusin, followed by a cell viability assay using the flow-cytometry method. Mean ± s.d. n = 3 replicates.

Figure S6

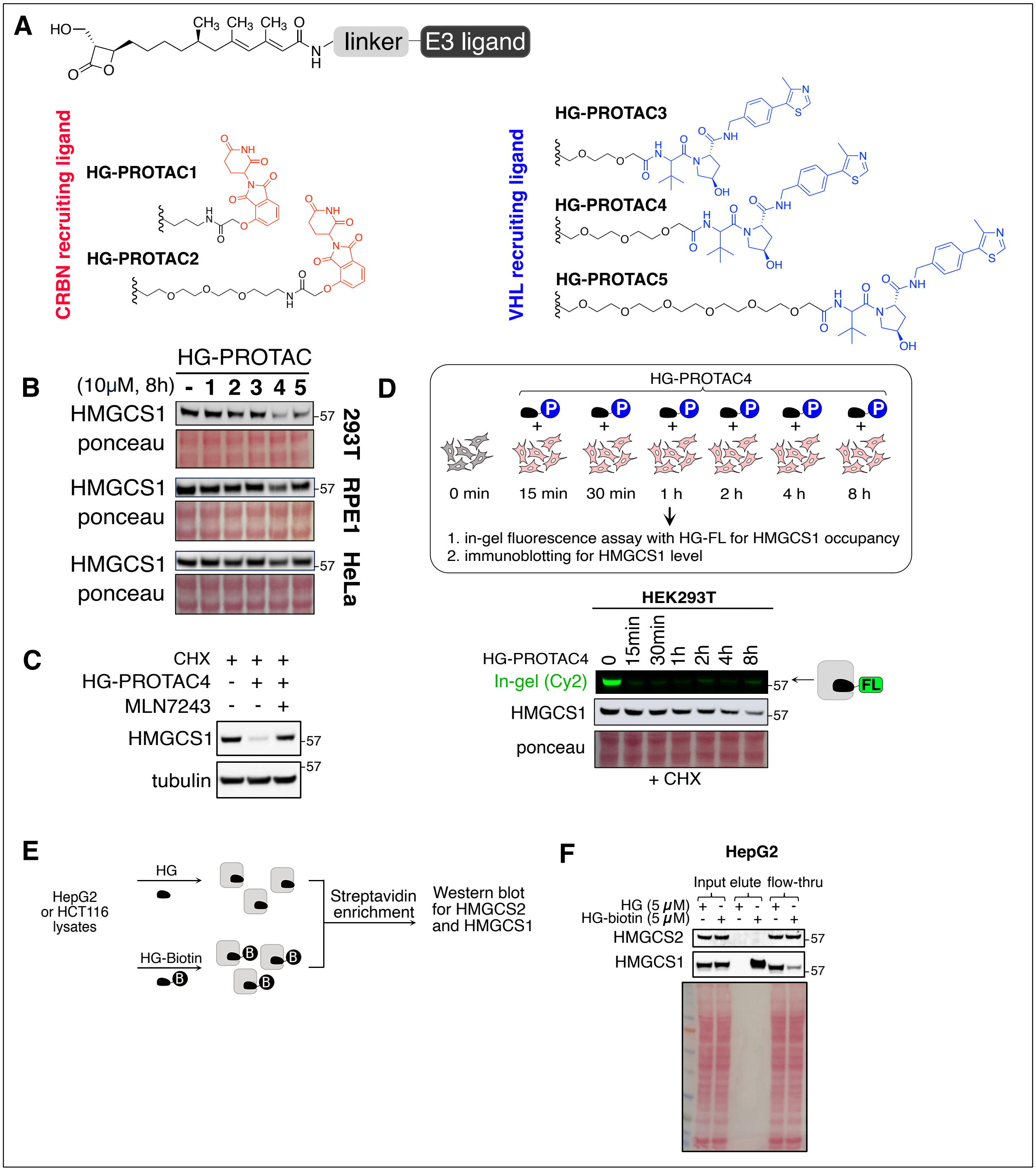

SI FIGURE 6.

(A) A framework of the Hymeglusin-PROTAC molecules is shown at the top. Structures of the five synthesized Hymeglusin-PROTAC candidates are displayed at the bottom. The CRBN-recruiting ligand or the VHL-recruiting ligand was conjugated using different linkers.

(B) HEK293T, RPE1, or HeLa cells were treated with each HG-PROTAC molecule (10 μM) for 8 hours. Cycloheximide (1 μM) was added to decouple the protein degradation from synthesis.

(C) HEK293T cells were treated with HG-PROTAC4 (10 μM, 16 h) in the presence or absence of ubiquitin-activating E1 enzyme inhibitor, MLN7243 (100 nM, 16 h).

(D) HG-PROTAC4-treated HEK293T cells in the presence of cycloheximide (1 μM) were collected at the indicated time points, and the lysates were treated with HG-FL to measure the occupancy of the catalytic cysteine of HMGCS1. After in-gel fluorescence analysis, the gel was processed to measure HMGCS1 levels by immunoblotting. Ponceau is shown as a loading control.

(E) Workflow for testing the reactivity of Hymeglusin toward HMGCS2.

(F) Immunoblotting analysis of the HepG2 lysates prepared as in panel E shows that HMGCS2 was not pulled down with HG-biotin, in stark contrast to HMGCS1 in the given condition.
