# Supplementary material for: Activity-based probes and chemical proteomics uncover the biological impact of targeting HMG-CoA Synthase 1 in the mevalonate pathway
Source: J Biol Chem. 2025 Sep 3;301(10):110660. doi: 10.1016/j.jbc.2025.110660 (PMC12514577; doi:10.1016/j.jbc.2025.110660)
Supplement: Supporting Table S3 [file mmc5.docx]

**Table S3. Key resources table**

| **REAGENT OR RESOURCE** | **SOURCE** | **IDENTIFIER** |
| --- | --- | --- |
| **Antibodies** | | |
| HMGCS1 (1:2000) | Cell Signaling Technology | 36877S; RRID:AB_2799107 |
| HMGCR (1:1000) | Abcam | Ab242315 |
| HMG-Lysine (1:500) | Sigma-Aldrich | ABS2108 |
| Tubulin, alpha (1:3000) | Abcam | Ab7291; RRID: AB_2241126 |
| RhoA (1:2000) | Cell Signaling Technology | 2117T; RRID: AB_10693922 |
| **Bacterial and virus strains** | | |
| E. coli DH5α | Enzynomics | CD011 |
| **Chemicals, peptides, and recombinant proteins** | | |
| REVERTTM Total Protein Stain Kit | LI-COR | P/N926-11010 |
| TCEP | Gold Biotechnology | TCEP10 |
| EPPS | Sigma-Aldrich | E9502 |
| 2-Chloracetamide | Sigma-Aldrich | C0267 |
| Protease inhibitor cocktail | Sigma-Aldrich | P8340 |
| PhosSTOP | Sigma-Aldrich | 4906845001 |
| Hymeglusin | Cayman Chemical | 11899 |
| Simvastatin | Millipore Sigma | PHR1438 |
| dTAGv1 | Tocris Bioscience | 6914 |
| GGTI-298 | Selleckchem | S7466 |
| Crystal violet | Sigma-Aldrich | HT901-8FOZ |
| **Critical commercial assays** | | |
| E.Z.N.A.® Plasmid DNA Mini Kit I | Omega Bio-tek | D6942-02 |
| GeneJET Gel Extraction Kit | Thermo Scientific | K0692 |
| **Experimental models: Cell lines** | | |
| Human: HEK293T | ATCC | CRL-3216; RRID:CVCL_0063 |
| Human: HCT116 | ATCC | CCL-247; RRID:CVCL_0291 |
| Human: HeLa | ATCC | CCL-2; RRID:CVCL_0030 |
| Human: RPE1 | ATCC | CRL-4000; RRID:CVCL_4388 |
| Human: MCF7 | ATCC | HTB-22; RRID:CVCL_0031 |
| Human: HepG2 | ATCC | HB-8065; RRID:CVCL_0027 |
| Human: U2OS | ATCC | HTB-96; RRID:CVCL_0042 |
| Human: MFE296 | Gift from the Neal Rosen lab | RRID: CVCL_1406 |
| Human: DLD1 | Gift from the Alicia M Pickrell lab | RRID: CVCL_0248 |
| Human: HEK293T overexpressing HMGCS1-V5 | Yi et al., Mol Cell. (2024) 84:2166-84. | Ref. 22 |
| Human: HEK293T overexpressing HMGCS1 (C129A)-V5 | This paper | N/A |
| Human: HMGCS1-FKBP12 F36V-V5 KI HCT116 | Yi et al., Mol Cell. (2024) 84:2166-84. | Ref. 22 |
| Human: HMGCS1-mEGFP KI HCT116 | Yi et al., Mol Cell. (2024) 84:2166-84. | Ref. 22 |
| Human: Simvastatin-resistant HMGCS1-mEGFP KI HCT116 | This paper | N/A |
| **Experimental models:Organisms/strains** | | |
| nu/nu athymic mice (Charles River Laboratory) |  |  |
| **Oligonucleotides** | | |
| Guide RNA sequence targeting C-terminus of HMGCS1: 5’-GAACATTAAGATACTCTGTG-3’ | Yi et al., Mol Cell. (2024) 84:2166-84. | Ref. 22 |
| **Recombinant DNA** | | |
| pHAGE-HMGCS1-V5 | Yi et al., Mol Cell. (2024) 84:2166-84. | Ref. 22 |
| pHAGE-HMGCS1 (C129A)-V5 | This paper | N/A |
| pSMART-HMGCS1-FKBP12 F36V-V5 | Yi et al., Mol Cell. (2024) 84:2166-84. | Ref. 22 |
| pSMART-HMGCS1-mEGFP | Yi et al., Mol Cell. (2024) 84:2166-84. | Ref. 22 |
| **Software and algorithms** | | |
| Prism 9 | GraphPad | <https://www.graphpad.com/scientific-software/prism> |
| Image Lab 6.1 | Bio-Rad Laboratories | <https://www.bio-rad.com/en-us/product/image-lab-software?ID=KRE6P5E8Z> |
| Perseus | Tyanova et al., Nat Methods. (2016) 13:731-40. | <https://maxquant.net/perseus/> |
| **Instruments** | | |
| Orbitrap Eclipse Tribrid Mass Spectrometer | Thermo Fisher Scientific | FSN04-10000 |
